# Plant DNA barcodes and assessment of phylogenetic community structure of a tropical mixed dipterocarp forest in Brunei Darussalam (Borneo)

**DOI:** 10.1371/journal.pone.0185861

**Published:** 2017-10-19

**Authors:** Jacqueline Heckenhauer, Kamariah Abu Salim, Mark W. Chase, Kyle G. Dexter, R. Toby Pennington, Sylvester Tan, Maria Ellen Kaye, Rosabelle Samuel

**Affiliations:** 1 Department of Botany and Biodiversity Research, Faculty of Life Sciences, University of Vienna, Vienna, Austria; 2 Environmental and Life Sciences, Faculty of Science, University of Brunei Darussalam, Gadong, Brunei Darussalam; 3 Jodrell Laboratory, Royal Botanic Gardens, Kew, Richmond, United Kingdom; 4 School of Biological Sciences, The University of Western Australia, Crawley, Western Australia, Australia; 5 School of GeoSciences, University of Edinburgh, Edinburgh, United Kingdom; 6 Royal Botanic Garden Edinburgh, Edinburgh, United Kingdom; 7 Geography, University of Exeter, Exeter, United Kingdom; 8 Sarawak Forest Department, Kuching, Sarawak, Malaysia; 9 School of Biological Science, University of Aberdeen, Aberdeen, United Kingdom; Chinese Academy of Forestry, CHINA

## Abstract

DNA barcoding is a fast and reliable tool to assess and monitor biodiversity and, via community phylogenetics, to investigate ecological and evolutionary processes that may be responsible for the community structure of forests. In this study, DNA barcodes for the two widely used plastid coding regions *rbcL* and *matK* are used to contribute to identification of morphologically undetermined individuals, as well as to investigate phylogenetic structure of tree communities in 70 subplots (10 × 10m) of a 25-ha forest-dynamics plot in Brunei (Borneo, Southeast Asia). The combined matrix (*rbcL* + *matK*) comprised 555 haplotypes (from ≥154 genera, 68 families and 25 orders sensu APG, Angiosperm Phylogeny Group, 2016), making a substantial contribution to tree barcode sequences from Southeast Asia. Barcode sequences were used to reconstruct phylogenetic relationships using maximum likelihood, both with and without constraining the topology of taxonomic orders to match that proposed by the Angiosperm Phylogeny Group. A third phylogenetic tree was reconstructed using the program Phylomatic to investigate the influence of phylogenetic resolution on results. Detection of non-random patterns of community assembly was determined by net relatedness index (NRI) and nearest taxon index (NTI). In most cases, community assembly was either random or phylogenetically clustered, which likely indicates the importance to community structure of habitat filtering based on phylogenetically correlated traits in determining community structure. Different phylogenetic trees gave similar overall results, but the Phylomatic tree produced greater variation across plots for NRI and NTI values, presumably due to noise introduced by using an unresolved phylogenetic tree. Our results suggest that using a DNA barcode tree has benefits over the traditionally used Phylomatic approach by increasing precision and accuracy and allowing the incorporation of taxonomically unidentified individuals into analyses.

## Introduction

Understanding community assembly and processes that are responsible for community diversity, species differentiation, and coexistence are important in the face of rapid global ecosystem change [1]. Three mechanisms are often put forward as drivers of community assembly [1]: (1) niche-related processes, in which community assembly is influenced by competition [2] and/or abiotic filters [3], (2) neutral processes, in which species are ecologically equivalent [4, 5, 6], and (3) historical processes, which bring an evolutionary perspective into community ecology [7, 8]. The relative importance of these processes for the assembly of communities and coexistence of species has been often debated [1, 5, 6, 9, 10, 11, 12]. Quantification of the phylogenetic component of biodiversity has become important in studying community assembly [13, 14] and holds promise to resolve the controversy over the relative importance of neutral vs. niche-related processes [1]. Phylogenetic information permits an understanding of how communities have evolved through time [15] and is being used increasingly to answer questions of community assembly e.g. [1, 13, 14, 16, 17, 18]. Community phylogenetic structure can exhibit three basic forms, random, clustered and overdispersed [13], although these should be viewed as part of a continuum. In a phylogenetically clustered community, co-occurring species are more closely related than expected by chance. Conversely, a phylogenetically overdispersed community contains species that are more distantly related than expected by chance. In turn, these forms are used as a proxy to suggest underlying mechanisms of community assembly [14]. Phylogenetic clustering can hint at abiotic-driven assembly processes (habitat filtering), which is based on the fact that under a given set of environmental conditions, closely related species are more likely to be similar in abiotically adaptive traits (trait conservatism). In contrast, in phylogenetically overdispersed communities, biotic interactions (e.g. interspecific competition) may be important in structuring the local community e.g. [19, 20]. These biotic factors can include herbivores and pathogens [21, 22, 23] because they are often specialized for the chemistry of related plants and therefore host shifts in general tend to occur among plants of similar chemistry [24]. Consequently, sharing of herbivores and pathogens could limit the coexistence of closely related plants that are similar in morphology and chemistry but facilitate coexistence of more distantly related plants with different traits.

Community phylogenetics uses phylogenetic trees of co-occurring species within a community to calculate phylogenetic diversity statistics (e.g. phylogenetic diversity [25]), net relatedness index (NRI; [13]), and nearest taxon index (NTI; [13]). Rapid construction of a community phylogenetic tree is often achieved using the online interface Phylomatic [26], which trims a reference tree for plants (Angiosperm Phylogeny Group, APG) to taxa occurring in the community. However, the Phylomatic procedure often provides little or no resolution of relationships among closely related species or even genera [27]. Moreover, for analyses using Phylomatic, the correct identification of individuals is mandatory, and this is often lacking in species-rich tropical forests. DNA barcoding has a high potential to reduce the number of unidentified individuals. DNA barcoding, besides its application in species identification and discovery of cryptic species e.g. [28, 29, 30], has a potential role to play in community phylogenetics [31]. For example, by using DNA barcode sequences to generate a phylogenetic hypothesis for a local species assemblage of woody plants of a forest-dynamics plot, Kress *et al*. [32] investigated community assembly on Barro Colorado Island, Panama. Since then, DNA barcode sequences have been successfully applied in studying the phylogenetic community structure of forests and other ecosystems e.g. [32, 33, 34, 35, 36, 37, 38, 39, 40, 41]. For plants, portions of two plastid genes, *matK* and *rbcL*, have been recommended by the Consortium for the Barcode of Life (CBOL) Plant Working Group [42]. In addition, a third marker, the plastid intergenic spacer *trnH-psbA* was proposed [43, 44] and has been used in phylogenetic community structure analyses [32, 33, 34, 40]. A disadvantage of DNA barcode phylogenetic trees of a single community is that due to sparse taxon sampling across the whole angiosperm tree (missing many families, genera, and species), they can be incongruent in topology with the accepted Angiosperm Phylogeny Group (APG) classification [45, 46]. Therefore, recently published studies e.g. [33, 39, 40] used the ordinal-level topologies of the Phylomatic tree as constraints in phylogenetic analyses of the barcoding data. This allows resolution of the tips of the Phylomatic tree while the deeper APG relationships are retained.

In contrast to Neotropical forests, where phylogenetic clustering is consistently reported as the predominant pattern [35, 47], most of the Southeast Asian forests are dominated by one particular angiosperm family, Dipterocarpaceae [48]. Therefore, interactions between close relatives that might promote overdispersion may be more important in structuring Southeast Asian forests. Patterns of phylogenetic community structure and phylodiversity have been investigated in a Southeast Asian forest before [13, 49] but using phylogenetic trees generated via the Phylomatic procedure that does not resolve relationships among genera or among species within genera, which is particularly important for detecting overdispersion. To date, no studies have been conducted on the phylogenetic structure of tree communities in Southeast Asia using DNA barcode sequences. Thus, such an analysis is imperative because the pattern of community structure may contrast with the existing view that phylogenetic clustering is paramount in tropical rain forests.

In this study, we assessed the phylogenetic structure for 70 subplots (10 × 10 m) within a 25-ha (500 × 500 m) of mixed dipterocarp forest in Kuala Belalong, Temburong, Brunei Darussalam, on the island of Borneo. An earlier study of a 1 ha plot in the same area as the research plot revealed the presence of 231 tree species [50]. As identification is ongoing, the exact number of species is still unknown, but estimates range between 850–1050 species across the 25-ha, making it among the most species-rich plots in Indomalayasia [51]. This high species-richness, much of which is contributed by species from species-rich genera (i.e. *Shorea*, *Syzygium*, and *Diospyros*) makes the Kuala Belalong plot an ideal location to assess the utility of DNA barcode sequences in a community phylogenetic study.

In this paper, we address the following questions:

1. Do the standard DNA barcodes (*rbcL* and *matK*) contribute to identification of morphotaxa occurring in the 70 subplots of the 25-ha forest-dynamics plot? We predict that the combination of conserved (*rbcL*) and a rapidly evolving (*matK*) barcoding regions allows identification of morphotaxa at least to genus-level if their sequences are already available in reference databases [52, 53], including the contributions to these from this study.
2. Does a community analysis based solely upon *rbcL* and *matK* barcoding sequence data offer significant benefits over one based on a phylogenetic tree constructed using Phylomatic? We expect that the high resolution predicted in the barcode tree decreases the bias and noise in NTI and NRI values, which have been commonly observed with Phylomatic trees due to a decrease in phylogenetic resolution [32].
3. What are the patterns of phylogenetic community structure in this forest and what do they tell us about drivers of community assembly? We suggest that Southeast Asian forests may show greater phylogenetic overdispersion than Neotropical forests because they are often disproportionately dominated by one clade of trees (in most cases, Dipterocarpaceae), thus increasing the general intensity of interspecific competition [14, 54]. In addition, the Bruneian research plot receives a high mean annual precipitation (5203 mm per year), which could allow for more natural enemies (pathogens) such as bacteria, fungi, and viruses that can promote phylogenetic overdispersion [21, 22].

## Material and methods

### Study site and sampling

All necessary permissions for this study were obtained in agreement with all relevant guidelines and policies as outlined in the collaboration agreement between Institute for biodiversity and environmental research (IBER), Universiti Brunei Darussalam and University of Vienna, Austria. The Biodiversity Research and Innovation Centre (BIORIC), Ministry of Industry and Primary Resources Brunei Darussalam granted export of biological specimens for research purposes under reference number BioRIC/HOB/TAD/51–30 and BioRIC/HOB/TAD/51–46.

The study was conducted in a long-term forest-dynamics plot (latitude: 4.634, longitude: 115.228, http://www.ctfs.si.edu/site/Kuala+Belalong, last accessed: 2017-08-19) that was established at the Kuala Belalong Field Studies Centre (KBFSC) of Universiti Brunei Darussalam in 2009 following the protocols of Condit [55]. It is part of the Center for Tropical Forest Science–Forest Global Earth Observatory (CTFS-ForestGEO; [50]) that includes 63 large-scale demographic tree plots across the Americas, Africa, Asia, and Europe, focusing mainly on the tropics [56]. The Bruneian plot is located in a primary, mixed dipterocarp forest in the Batu Apoi Forest Reserve at Temburong. This region is characterized by a tropical climate with significant year-round mean annual precipitation of 5203 mm and a mean annual temperature of 26.5°C [51]. It has a steep topography and elevation ranging from 160 to 320 m. The dominant soils are silty clay dominated by quartz and kaolinite (ultisoils). Besides being high in iron and aluminium oxides, they are extremely low in basic plant nutrients [50]. The natural disturbance regime is characterized by landslides [57]. The plot is dominated by broadleaf evergreen vegetation. The 25-ha plot is divided into 2500 subplots of 100 m^2^. All free-standing woody stems ≥ 1 cm diameter at breast height are tagged with individual numbers, measured, and mapped spatially. Reference vouchers are deposited at the University of Brunei Darussalam Herbarium (UBDH), and the tagged stem itself serves as an additional living voucher for the individuals sampled. Morphological identifications of the individuals are on-going. Following the CTFS standard protocol, specimens have been sorted to families, genera, and “morphospecies” and in this case have been identified by author the S. Tan. However, these “morphotaxa” have not yet been verified by comparison with vouchers at all pertinent herbaria, and a large number remain unidentified to species level. Across the 25-ha plot, 70 subplots (100 m^2^ each) were selected in a stratified random pattern including different topographical attributes. According to the list of individuals provided by UBD-CTFS, there are 4348 tagged trees in the 70 subplots. However, several tree tags were not found during sampling (presumably fallen off or removed by people). Leaf or bark material was sampled only from tagged individuals, leading to 3930 samples, which were dried in silica gel [58].

### Topographical analyses

**Fig 1:**
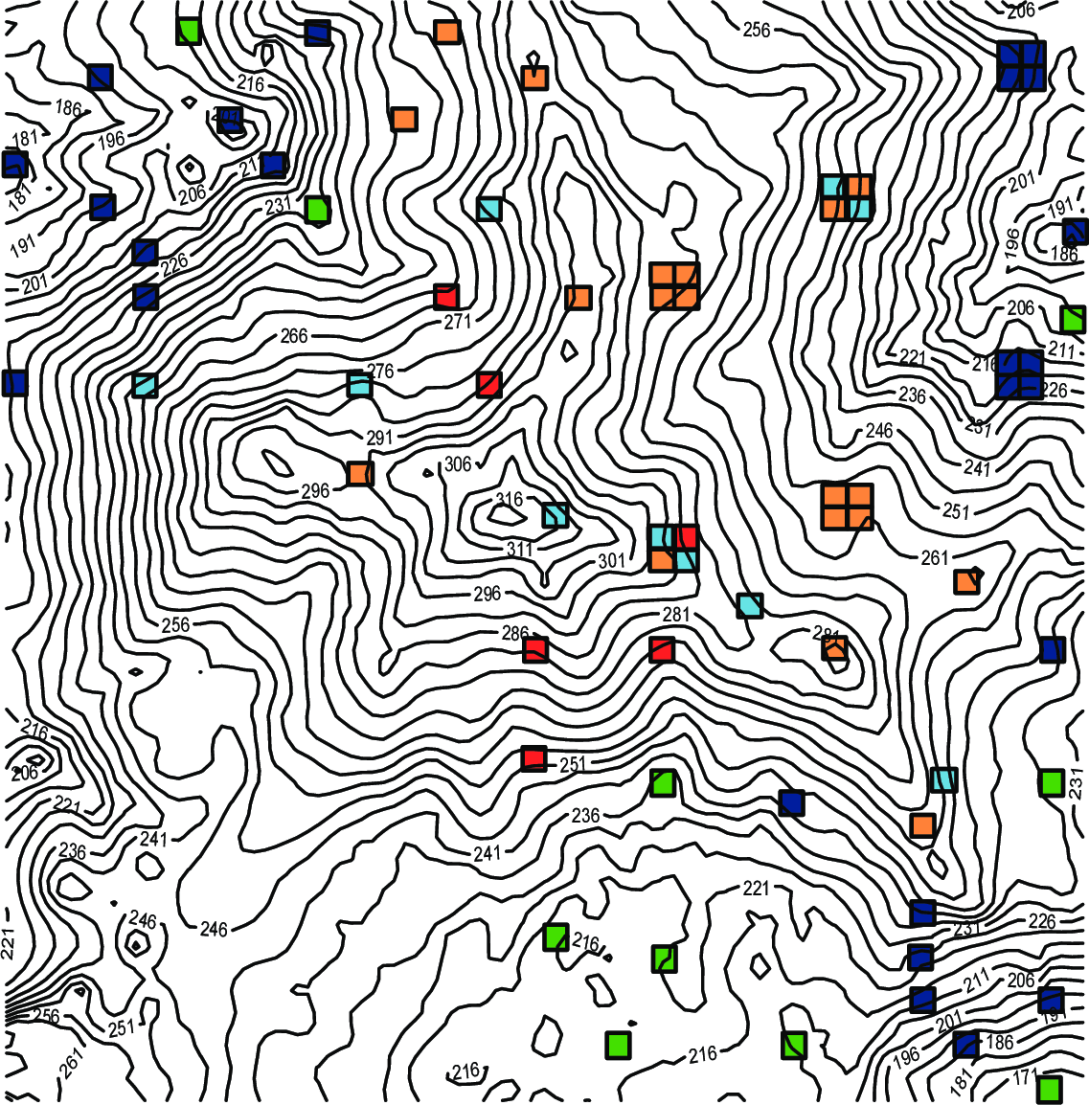
Contour map of the 25 ha plot in Kuala Belalong-Brunei Darussalam and location of the 70 subplots sampled in this study. Habitat types are given for each subplot: valley (green), low slope (dark blue); high slope (light blue); high gully (red); ridge top (orange).

Topographical raw data were provided by the UBD-CTFS and generated following standard protocols described by Condit [55]. Using the CTFS R package [59] a contour map was constructed (Fig 1). Three topographical parameters were calculated for each subplot (S2 Table): elevation (E), slope (S), and convexity (C). Elevation was defined as the mean elevations at the four corners of each quadrant [60]. Following Yamakura *et al*. [61], the convexity of each subplot was determined by calculating the difference of the mean elevation of the focal quadrat and mean elevation of 12 points along a grid of eight subplots surrounding the focal quadrat. For subplots located at the edge of the 25-ha plot, convexity was the elevation of the center point minus the mean of the four corners. Convex surfaces are indicated by positive values, whereas negative values indicate concave surfaces. Slope was calculated for each subplot using the quadslope function of the CTFS R package [62]. The three topographical variables were used to assign each of the 70 subplots to one of five habitats according to earlier studies e.g. [63]. These habitat types are (Fig 1, Table 1): valley (S < S*mean*, E < E*mean*); low slope (S ≥S*mean*, E < E*mean*); high slope (S ≥ S*mean*, E ≥ E*mean*, convexity > 0); high gully (S ≥ S*mean*, E ≥ E*mean*, convexity < 0); ridge top (S≤ S*mean*, E ≥ E*mean*).

**Table 1:** Habitat classification.

	Habitat				
	High gully (hg)	High slope (hs)	Low slope (ls)	Ridge top (rt)	Valley (v)
**Number of plots**	6	10	25	19	10
**Slope (°)**	≥ 27.5	≥ 27.5	≥ 27.5	≤ 27.5	< 27.5
**Elevation (m)**	≥ 243.5	≥ 243.5	< 243.5	≥ 243.5	< 243.5
**Convexity (°)**	< 0	> 0	All	all	all

Criteria (slope, elevation, and convexity) used for habitat classification are given.

### DNA barcode reference database and identification of morphologically unidentified individuals

#### DNA extraction, PCR amplification and sequencing

Prior to DNA extraction, samples were frozen in liquid nitrogen and ground into fine powder. Subsequently, genomic DNA was extracted from approximately 20 mg of material using the DNeasy 96 Plant Kit (QIAGEN, Hilden, Germany) following the manufacturer’s protocol. Working stocks of 10× diluted DNA were prepared. In total 3300 individuals were included. Two coding plastid regions, *rbcL* and *matK*, were amplified. For amplification of the *rbcL* region primers rbcLa_f [64] and rbcL 724R [65] were used. PCR reactions included 5 μL of 2× ReddyMix PCR Master Mix with 1.5 mM MgCl_2_ (#AB-0575/DC/LD/A; Thermo Fisher Scientific, Vienna, Austria), 0.1 μl 4.0% bovine serum albumin, 0.1 μl each primer (0.32 μM), 1 μl template DNA and H_2_0 up to a final volume of 10 μl. Thermal cycle conditions were as follows: initial denaturation at 98°C for 30 sec, 35 cycles of denaturation at 98°C for 10 sec, annealing at 63°C for 30 sec and extension at 72°C for 30 sec, followed by final extension of 5 min at 72°C. At the beginning of the study, there were three frequently used *matK* primer pairs available to amplify approximately the same region of the gene: 390F and 1326R [66, 67], XF and 5R [68], and 1R_KIM and 3F_KIM [42, 69]. Initially, all three primer pairs were used in this study following the authors’ protocols. In the course of generating *matK* sequences, a universal set of primers that can be multiplexed in one PCR reaction was developed (C_MATK_F and C_MATK_R, [70]). This set of primers was then used as follows: 5 μL of 2× ReddyMix PCR Master Mix with 1.5 mM MgCl_2_ (#AB-0575/DC/LD/A; Thermo Fisher Scientific, Vienna, Austria), 0.1 μL of forward and reverse primer cocktail each at 50 μM (final concentration 0.5 μM), 1 μL of template DNA, and H_2_O up to a final volume of 10 μL. Thermocycler conditions were as follows: 95°C for 2 min: five cycles of 95°C for 25 s, 46°C for 35 s, and 70°C for 1 min; 35 cycles of 95°C for 25 s, 48°C for 35 s, and 70°C for 1 min; and a final extension at 72°C for 5 min. For samples that did not amplify using the above-mentioned protocol, the 2× Phusion Green HS II Hi-Fi PCR Master Mix with 1.5 mM MgCl_2_ (#F-566S, Thermo Fisher Scientific, Vienna, Austria) was used with the following thermocycler conditions: 98°C for 30 s; five cycles of 98°C for 10 s, 53°C for 30 s, and 72°C for 30 s; 35 cycles of 98°C for 10 s, 55°C for 30 s, and 72°C for 30 s; and a final extension at 72°C for 5 min. PCR products were cleaned with 1.5 μL exonuclease I and FastAP thermosensitive alkaline phosphatase mixture (7 U Exo I, 0.7 U FastAP, Thermo Fisher Scientific, Vienna, Austria) at 37°C for 45 min and 85°C for 15 minutes. Sequencing reactions were performed with the BigDye Terminator Kit v3.1 (Thermo Fisher Scientific, Vienna, Austria) using the amplification primers according to the manufacturer’s instructions. Sanger sequencing was carried out using a 3730 DNA analyzer (Thermo Fisher Scientific, Vienna, Austria) at the Department of Botany and Biodiversity Research, University of Vienna.

#### Sequence assembly, editing, and alignment

Bidirectional sequences were trimmed, assembled into contigs, and edited in Geneious (version 8.0.5, [71]). Edited sequences were checked for contamination using BLAST [72]. Contaminated sequences, as well as samples that failed to produce quality reads for *matK* and *rbcL* were removed from the dataset, leading to a total of 3118 sequences for *rbcL* and 2598 sequences of *matK*. A local reference database for taxa occurring in the 70 subplots of the 25-ha plot was built by uploading all sequences to the Barcode of Life Datasystem [53] under code DS-PCSBRU1. Sequences were sorted according to their haplotypes by aligning them with MAFFT version 7.017 implemented in Geneious version 8.0.5 [71]. A representative for each haplotype was blasted against the Barcode of Life reference (BOLD) database [53] as well as to the National Center for Biotechnology Information (NCBI) reference database Genbank [52]. The resulting identifications were compared with the preliminary morphological identifications. Morphologically unidentified individuals were identified to family or generic level according to their DNA sequence. To decrease computation time in subsequent analyses, a pruned data matrix using one representative per haplotype and morphotaxon was used. If the same morphotaxon exhibited different haplotypes, a representative for each haplotype was included (S1 Table). Due to the absence of indel variation, *rbcL* sequences were aligned directly in BioEdit v.7.0.4 [73]. Following translation into amino acids, *matK* sequences were aligned in BioEdit. The translated *matK* matrix was then edited manually. Both alignment files for each marker were combined. For analysis, unsequenced regions and gaps were coded as missing data.

### Reconstruction of phylogenetic community trees

To compare resolution and node support of different phylogenetic approaches, three trees were constructed in this study. A tree based on the most recent reference tree R20120829 (APG III, [45]) was built using the online version of Phylomatic [26]. For this, a list of taxa occurring in the barcode matrix was submitted to the program, which tries to match the taxa to the most resolved position in a stored tree. This rapid phylogenetic reconstruction represents a classic and widely used approach in community phylogenetics [74, 75, 76]. Trees were also inferred from the barcode data. Substitution rates were estimated independently for each gene. Here, the rapid bootstrapping algorithm (1000 replicates), which does a complete analysis (ML search and bootstrapping) in one step was conducted using RaxML v8.2.0 [77]. The general time reversible model with six substitution rates (one for each pair of nucleotides) and gamma-distributed rate variation across sites (GTRGAMMA) was chosen for the analysis based on jModeltest2 [78]. The tree constructed by Phylomatic mostly resolves relationships at family level, whereas the barcode data helps to resolve relationships at generic or even species-level. An additional analysis was conducted here because deep nodes in a community phylogenetic tree based on barcodes may not resolve relationships correctly because of taxon-sampling issues. To correct this, deep-level phylogenetic relationships were fixed using a constraint tree based on the APG classification e.g. [33, 39, 40] and the terminal tips were resolved using the barcode sequences. This constraint tree was built using the package “ape” [79] with the R programming language. All taxa were present in the constraint tree, but within each order species were arrayed as polytomies. The constraint tree was implemented in a RaxML analysis as described above, and only trees concordant with ordinal relationships of the APG tree were retained (S1 Text).

For phylogenetic community structure analyses, ultrametric trees are normally used. For the Phylomatic tree, this is typically done using the command “bladj” in Phylocom [80]. This command was used to obtain a pseudo-chronogram with adjusted branch lengths based on the node calibrations of Wikström *et al*. [81]. Both the unconstrained as well as the constrained trees obtained from the maximum likelihood analyses were transformed into ultrametric chronograms with the mean-path-length method (MPL, [82]) in PATHd8 [83] using age constraints of Magallón & Castillo [84]. They included one fixed age for the angiosperm crown group and 28 (unconstrained tree, S2 Text) or 29 (constrained tree, S3 Text) minimal age estimates.

### Phylogenetic community structure analyses

To enable a direct comparison among the phylogenetic approaches (Phylomatic and the two ML analyses with barcode sequences, unconstrained and constrained), all three chronograms were used to quantify the phylogenetic structure of 70 communities in the 25-ha forest research plot. If species showed more than one haplotype, we aimed at sequencing all individuals of those species in the plot to assign them to a single tip in the phylogenetic tree. Representatives (3241 individuals in total) for most of the morphotaxa were sequenced. Based on the assumption that the individuals of the same morphotaxon will have identical sequences for *matK* and *rbcL*, unsequenced individuals with a morphological identification were assigned to the haplotype (i.e. tip in the tree) corresponding to sequenced individuals with the same morphological identification. Thus, only taxa that lacked either morphological or sequence information (only 3.3% of the total number of individuals) were excluded from the community data matrix (S4 Text). In order to determine if our results were consistent without making this assumption, we repeated phylogenetic community structure analyses using only sequenced individuals (75% of individuals in the 70 communities). Results of this analysis are referred to as “Barcode only” in the text. In this sensitivity analysis, only 68 subplots were included, as in two subplots, most individuals lacked sequences. The reduced community data matrix with only sequenced individuals is given in S5 Text. Common phylogenetic diversity metrics were estimated with the remaining data using the package “picante” [85] in R. The widely used quantitative measure of phylogenetic diversity, PD, [25] was calculated on the basis of a chronogram using the “pd” function. In this approach, the branch lengths of a phylogenetic tree, in units of time, are measured and summed. To compare each of the three trees, PDs were compared for subplots using a paired t-test. The phylogenetic trees were then converted into an interspecific phylogenetic distance matrix using the “cophenetic” function in "picante”. Based on this distance matrix, mean pairwise distance (MPD; [14]) and mean nearest taxon distance (MNTD; [86]) were calculated. The function “mpd” calculates the mean pairwise distance between all species or individuals in each community, and “mntd” calculates the mean nearest taxon distance, the average distance separating each species or individual in the community from its closest heterospecific relative. MPD and MNTD were weighted by species abundance. Using the functions “ses.mpd” and “ses.mntd”, a standardized effect size (SES) of the metric within each local community was calculated based on a comparison of observed MPD/MNTD (obs) values with the distribution of MPD/MNTD expected under a null model of community assembly where subplots have the same species richness, but species identities are randomised by randomly shuffling tip labels across the entire tree (rand; number of randomizations: 1000). To test for phylogenetic clustering and overdispersion, the net relatedness index (NRI) and the nearest taxon index (NTI) were calculated [13]. NRI and NTI are defined as [-(metric_obs_−mean (metric_rand_))/sd (metric)_rand_], where the metric is either MPD (for NRI) or MNTD (for NTI). Thus, they are equivalent to the inverse of ses.MPD and ses.MNTD. Positive indices indicate that co-occurring species are more closely related than expect by chance (phylogenetically clustered), whereas negative indices indicate that co-occurring species are more distantly related than expected by chance (phylogenetically overdispersed). NRI and NTI were compared between the different habitats. To investigate if there is a correlation between the environmental variables (mean elevation, slope, convexity) and community structure metrics (PD, NRI, NTI), Pearson product-moment correlation tests were conducted.

## Results

### DNA barcode reference database and identification of morphologically unidentified individuals

#### DNA barcode sequence recovery and abundance of families

**Fig 2:**
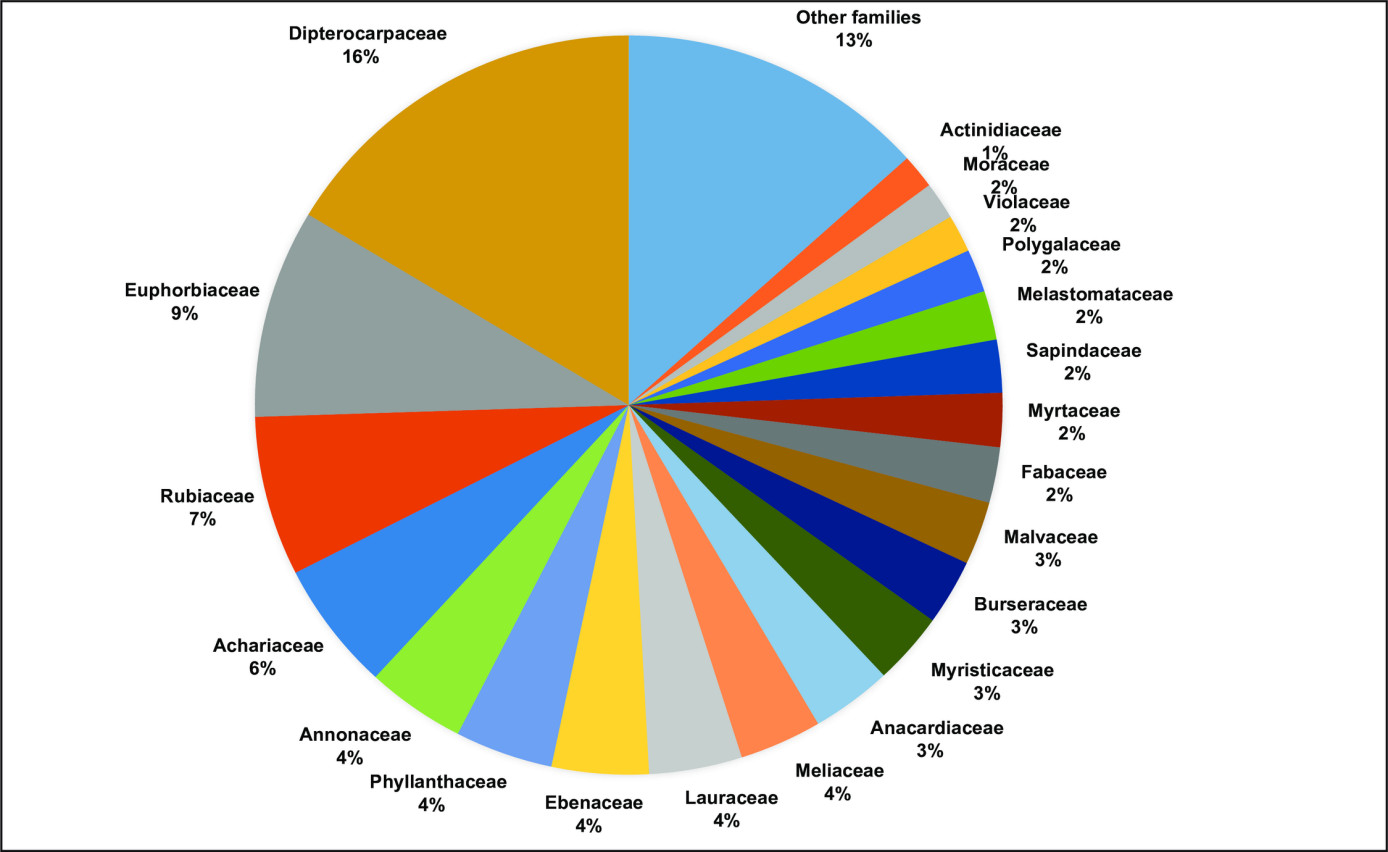
Abundance of plant families. Abundance of frequent plant families in the 70 subplots of the 25 ha forest dynamics plot in Kuala Belalong.

A DNA barcode reference database was successfully built for individuals occurring in the studied subplots of the 25-ha forest-dynamics plot. In total, DNA barcode sequence data was successfully recovered from 95.5% (*rbcL*), 78.7% (*matK*), and 71.6% (*rbcL + matK*) of sequenced individuals. The combined data matrix represented 555 haplotypes (from ≥ 154 genera, 68 families, 25 orders). The DNA barcode sequences were useful for determinations of taxa morphologically unidentified to family or genus, which is necessary for inclusion in phylogenetic reconstruction using Phylomatic. For 500 morphological unidentified individuals, DNA barcodes gave clear identification at genus or family level. Among the 69 families detected by both morphology and molecular identification, Dipterocarpaceae and Euphorbiaceae are dominant, with 16% and 9% of stems, respectively in the study plot. Other frequent families were Rubiaceae (7%) and Achariaceae (6%). The most abundant families are shown in Fig 2.

#### Characteristics of the alignments

The two-gene alignment included a total of 1820 base pairs (bp), 697 bp from *rbcL* and 1123 from *matK*. The number of variable characters of the combined data matrix was 1087, and the proportion of gaps and completely undetermined characters was 21.17%. Variable characters observed for each marker were 304 (*rbcL*) and 783 (*matK*). The number of gaps and undetermined characters were 0.54% for *rbcL* and 26.68% for *matK*. Population-level variation was detected in one or both loci for only 15 “morphospecies”. Additionally, six taxa (*Koompassia excelsa* and five species of *Xanthophyllum*) exhibited stop codons in the *matK* barcode region and were therefore classified as pseudogenes, but they were included in the analysis because these taxa fell in phylogenetic positions reflecting their taxonomy.

### Reconstruction of phylogenetic community trees

**Fig 3:**
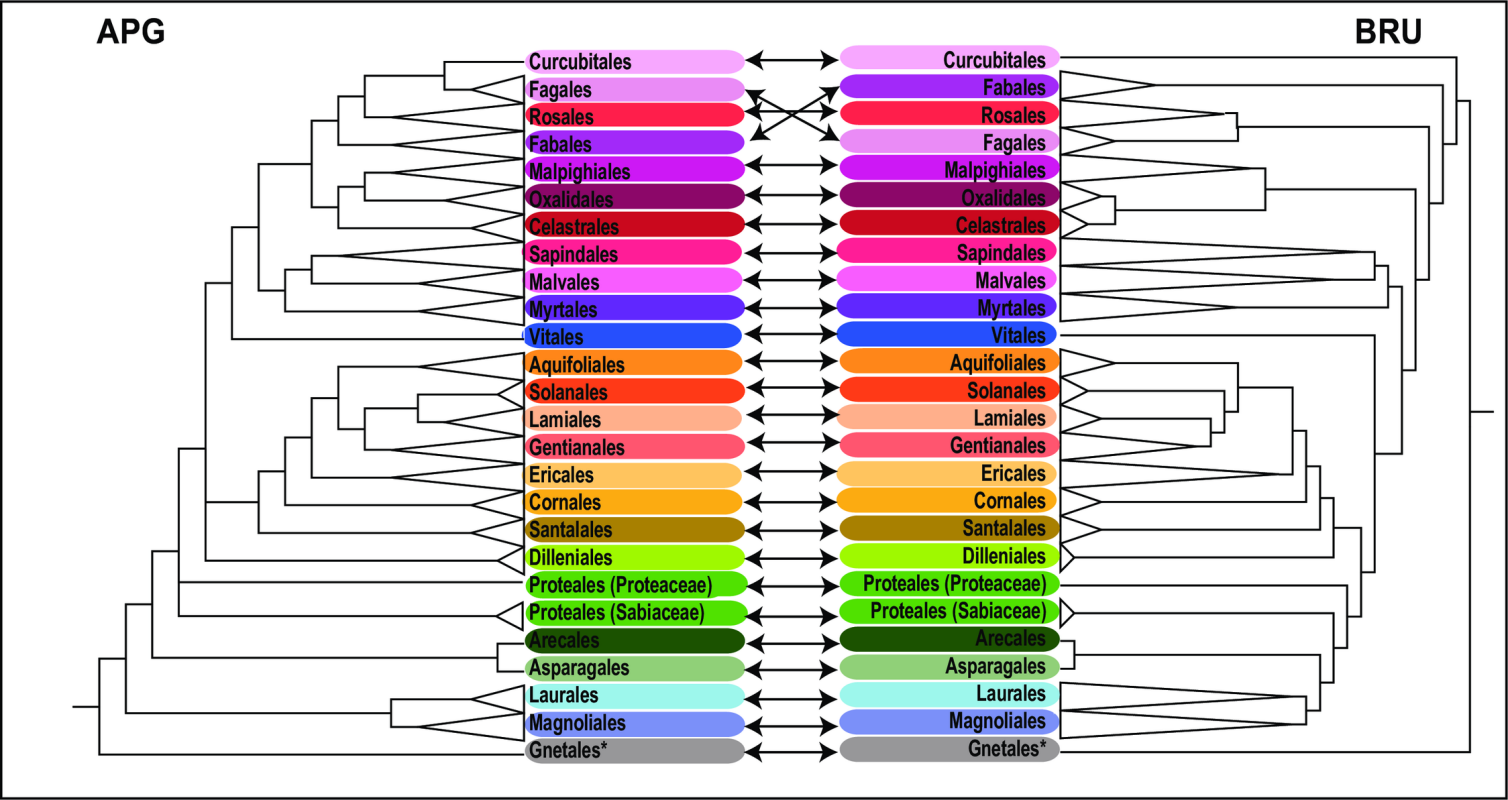
Comparison of ordinal-level topologies of the trimmed APG tree obtained by Phylomatic (APG) and the barcode tree (*rbcL* + *matK*; BRU) obtained from maximum likelihood analysis. Orders are connected by arrows. *: The order Gnetales represents the gymnosperms, whereas all other orders are angiosperms.

**Fig 4:**
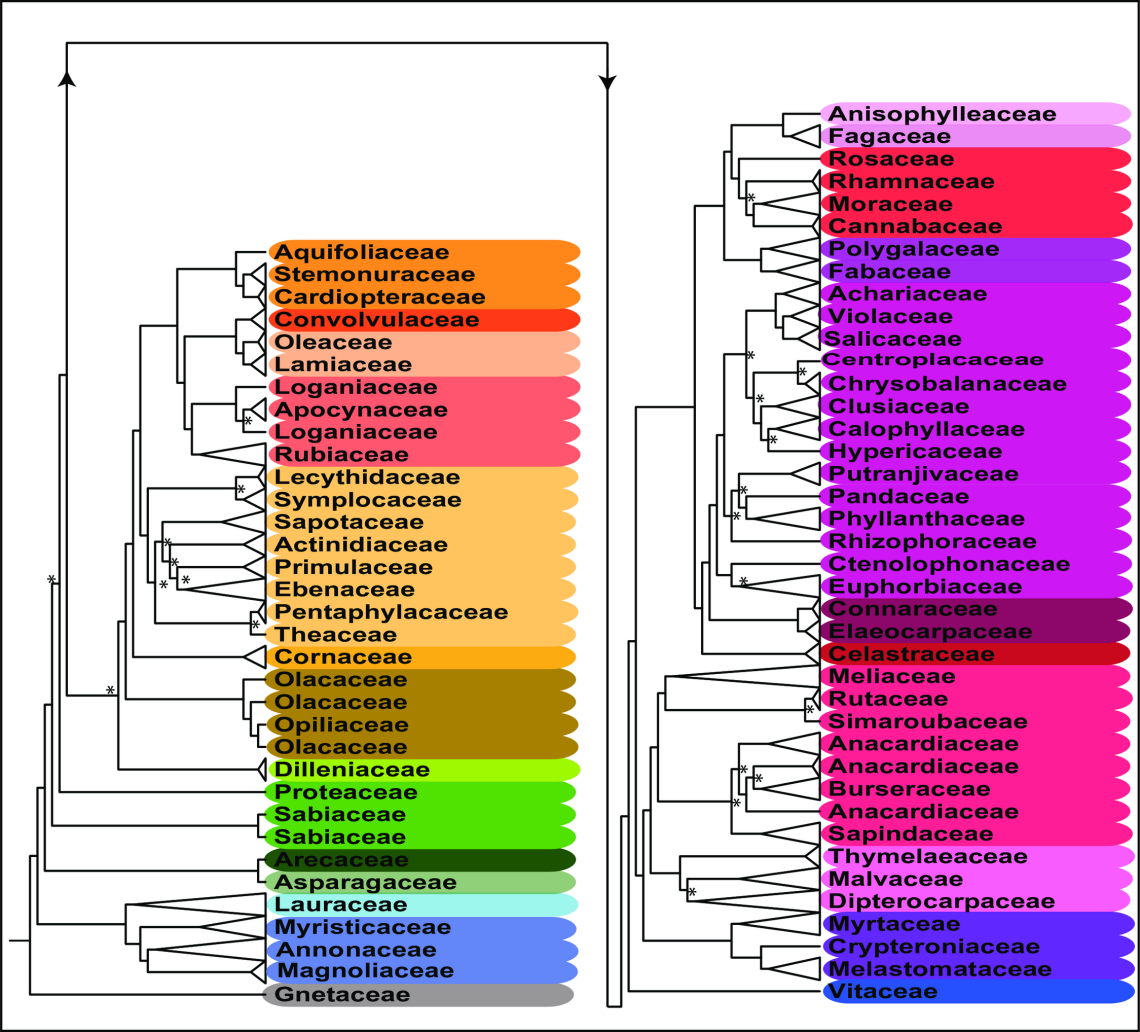
Cladogram of phylogenetic relationships of the woody plant taxa in the Kuala Belalong forest dynamics plot, Brunei Darussalam (BRU). Best-scoring tree obtained from maximum likelihood analysis of the barcode data (*rbcL*+*matk*) with application of an APG III-based ordinal-level constraint tree. The tree is collapsed to family level. For presentation purposes a cladogram is given. An uncollapsed tree including branch lengths is given in S1 Text. Nodes with an * have bootstrap support < 70.

All trees produced in this study are given in S1 Text. The trimmed APG reference tree (R20120829) obtained by Phlylomatic includes 186 resolved nodes, mainly at ordinal and family level, but in some cases resolving relationships among genera within families. Other than Proteales, all other orders were monophyletic. The two families of Proteales were unresolved, a result in common with many other analyses e.g. [87]. Bootstrap support (BS) for this placement was not strong in earlier studies ([88]: BS: 59; [89]: BS: 63), even with complete plastid genomes. In the ML tree constructed using the barcode data, 42.7% of the nodes exhibited high bootstrap support (BS > 85) and a majority (52.4%) showed at least moderate support (BS ≥ 70). Contrary to the Phylomatic tree, the DNA barcode markers were able to resolve relationships at all taxonomic levels, with better resolution at generic and especially species level. Examples of these fine-scale relationships are the genera *Diospyros* and *Shorea* for which species relationships remain completely unresolved in the Phylomatic tree (S1 Text). Furthermore, all families were grouped into the same orders as in APG III [45] and APG IV [46], Sabiaceae and Proteaceae (Proteales). However, the topology of the tree differed from the accepted APG classification at the ordinal level (Fig 3). The constrained tree successfully resolved relationships at all taxonomic levels. Compared to the Phylomatic and the unconstraint barcoding trees, the constrained tree showed the highest percentage of highly supported nodes (BS > 85: 43.6% and BS ≥ 70, 53.8%). Proteales were paraphyletic in the constrained analysis, and all other families clustered in the APG IV orders [46]. Three families, Olacaceae (Santalales), Anacardiaceae (Sapindales) and Loganiaceae (Gentianales) were not monophyletic, but monophyly of the remaining families was highly supported (BS ≥ 85), except for Euphorbiaceae (BS 46; Fig 4).

### Assessment of phylogenetic community structure

#### Phylogenetic diversity (PD)

**Fig 5:**
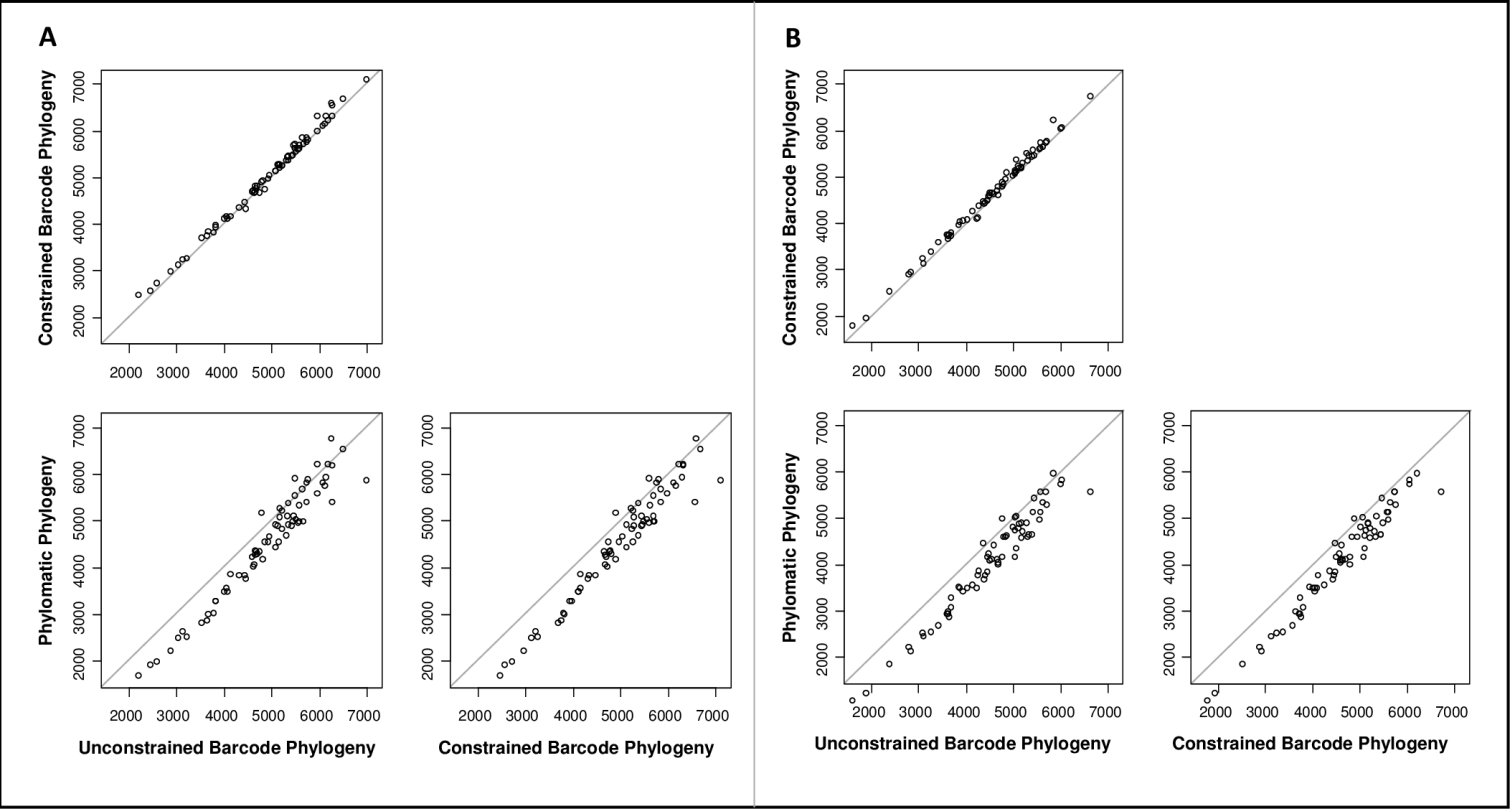
Pairwise comparison of phylogenetic diversity (PD). PD was calculated based on three different phylogenetic hypotheses (Phylomatic: APG classification, Barcode.con: constrained barcode tree, Barcode.uncon: unconstrained barcode tree). A: Calculations based on sequenced and morphologically identified individuals. B: Calculations based on sequenced individuals only.

Mean PD of the subplots varied among the different trees (Fig 5, S3 Table). It was highest when calculated based on a constrained ML tree (4980.46 myrs; Barcode only: 4479.02 myrs), followed by the unconstrained ML tree (4891.62 myrs; Barcode only: 4401.81 myrs) and the tree constructed using Phylomatic (4519.63 myrs: Barcode only: 3956.84 myrs). Using paired t-tests, differences in PD between the calculations based on the unconstrained and constrained ML analyses were significant (t = 8.7228, df = 69, p-value = 9.544e-13; Barcode only: t = 8.5322, df = 67, p-value: 2.646e-12). Highly significant differences were detected between calculations using Phylomatic and the unconstrained (t = -9.7575, df = 69, p-value = 1.271e-14; t = -14.686, Barocode only: df = 67, p-value < 2.2e-16), as well as the constrained (t = -12.365, df = 69, p-value < 2.2e-16; Barcode only: t = -17.857, df = 67, p-value < 2.2e-16) barcoding ML analyses (Fig 5).

#### Phylogenetic community structure

**Fig 6:**
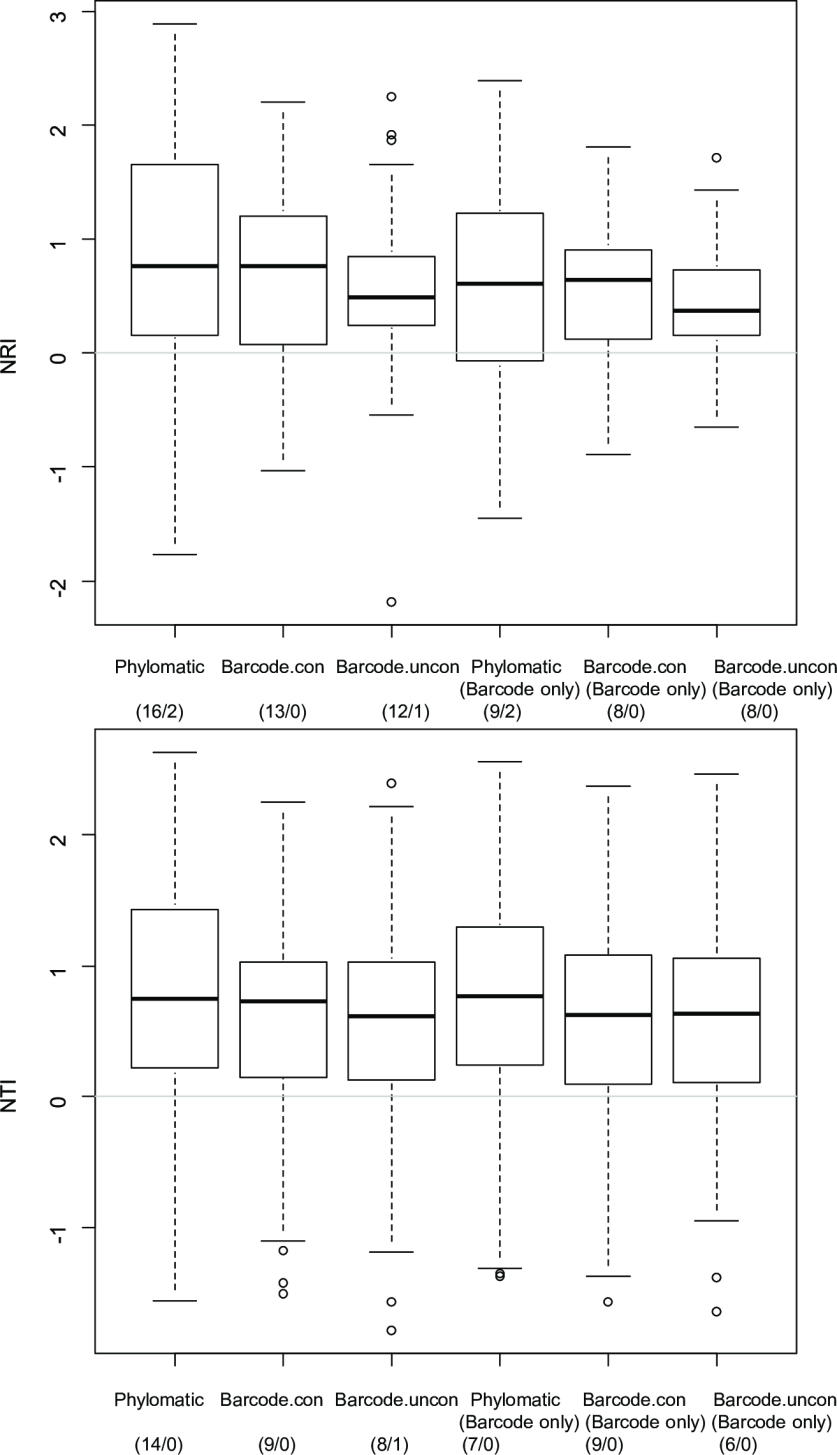
Net relatedness index (NRI) and nearest taxon index (NTI). NRI and NTI were calculated based on three different phylogenetic hypotheses (Phylomatic: APG classification, Barcode.con: constrained barcode tree, Barcode.uncon: unconstrained barcode tree). The number of subplots showing significant phylogenetic structuring (clustering/overdispersion) is given in brackets. A: Calculation based on sequenced and morphologically identified individuals. B: Calculations based on sequenced individuals only.

**Fig 7:**
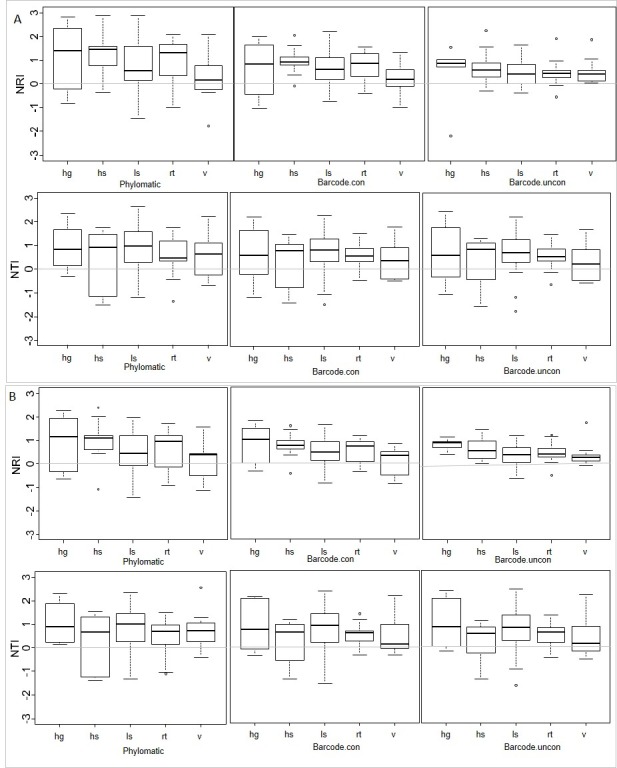
Comparison of net relatedness index (NRI) and nearest taxon index (NTI) in different habitats. NRI and NTI were calculated based on three different phylogenetic hypotheses (Phylomatic: APG classification, Barcode.con: constrained barcode tree, Barcode.uncon: unconstrained barcode tree) in different habitats (hg: high gully, hs: high slope, ls: low slope, rt: ridge top, v: valley). A: Calculation based on sequenced and morphologically identified individuals. B: Calculations based on sequenced individuals only.

Comparing the NRI and NTI metrics, similar patterns of phylogenetic community structure were observed. In some cases, patterns of phylogenetic community structure varied with respect to the tree used for calculation (Fig 6, S3 Table). Looking at the NRI metric of each subplot, the Phylomatic tree detected significant phylogenetic clustering in 16 subplots (Barcode only: nine) and significant phylogenetic overdispersion in two subplots (Barcode only: two). Using the barcode tree, significant clustering was detected in 13 (constrained, Barcode only: eight) and 12 (unconstrained, Barcode only: eight) subplots. Overdispersion was detected in one subplot using the unconstrained barcode tree. For the NTI metric, phylogenetic clustering was detected in 14 subplots (Barcode only: seven) using the Phylomatic tree, whereas the barcoding trees detected clustering in nine (constrained, Barcode only: nine) or eight (unconstrained, Barcode only: six) subplots. The unconstrained barcode tree revealed phylogenetic overdispersion in one subplot. Overall, the Phylomatic tree not only exhibited a higher mean for NTI and NRI, but also a much greater variance (variance of NRI: Phylomatic: 1.19 (Barcode only: 0.88), Barcode.con: 0.56 (Barcode only: 0.41), Barcode.uncon: 0.42 (Barcode only: 0.46); variance of NTI; Phylomatic 0.96 (Barcode only: 0.83), Barcode.con: 0.71 (Barcode only: 0.66), Barcode.uncon: 0.76 (Barcode only: 0.67)). A summary of subplots exhibiting significantly phylogenetic structuring with respect to different trees is given in S3 Table. Although there were differences in detecting phylogenetic structure with different phylogenetic trees and community data matrices, no reversed inferences for NRI and NTI were observed. Furthermore, phylogenetic clustering was detected in all habitats (Fig 7). To conclude, other than a few cases of significant phylogenetic overdispersion, the general pattern of either random structuring or phylogenetic clustering did not differ with respect to phylogenetic tree, habitat or community matrix (Fig 6, Fig 7).

#### Processes responsible for community assembly

Patterns of phylogenetic structuring are often used as a proxy for the mechanism responsible for community assembly. Phylogenetic clustering can suggest the influence of abiotic factors on community assembly [14]. Consequently, three environmental variables (mean elevation, slope, and convexity) were tested for correlations between PD, NRI, and NTI. Pearson product-moment correlations detected a weak positive correlation between the PD and mean elevation when calculated on basis of the unconstrained barcoding tree (Table 2). A moderate positive correlation between mean elevation and NRI was observed when calculated using the Phylomatic tree and the constrained barcoding tree. Significant, albeit generally weak, positive correlations between convexity and PD, as well as between convexity and NRI, were observed in analyses of all three phylogenetic trees. Analysis based on the barcode data only revealed a positive correlation between NRI and elevation, as well as between NRI and convexity when the Phylomatic and the constrained barcode tree were used, whereas the unconstrained barcode tree showed positive correlation between NRI and convexity only (Table 2).

**Table 2:** Results of Pearson moment-product correlation test.

**Environmental** **variable**	**Phylomatic**			**Barcode.con**			**Barcode.uncon**		
	**PD**	**NRI**	**NTI**	**PD**	**NRI**	**NTI**	**PD**	**NRI**	**NTI**
**Mean elevation**	r = 0.18	**r = 0.39**	r = 0.1	r = 0.23	**r = 0.35**	r = -0.09	**r = 0.23**	r = 0.11	r = - 0.07
	p = 0.13	**p < 0.05**	p = 0.41	p = 0.05	**p < 0.05**	p = 0.47	**p = 0.049**	p = 0.34	p = 0.54
**Slope**	r = 0.04	r = 0.11	r = 0.03	r = 0.04	r = 0.11	r = -0.02	r = 0.02	r = 0.05	r = 0.005
	p = 0.72	p = 0.35	p = 0.81	p = 0.77	p = 0.38	p = 0.87	p = 0.84	p = 0.66	p = 0.97
**Convexity**	**r = 0.38**	**r = 0.44**	r = 0.08	**r = 0.36**	**r = 0.46**	r = 0.13	**r = 0.37**	**r = 0.27**	r = 0.14
	**p < 0.05**	**p < 0.05**	p = 0.51	**p < 0.05**	**p < 0.05**	p = 0.29	**p < 0.05**	**p < 0.05**	p = 0.25
**Environmental** **variable**	**Phylomatic Barcode only**			**Barcode.con Barcode only**			**Barcode.uncon Barcode only**		
	**PD**	**NRI**	**NTI**	**PD**	**NRI**	**NTI**	**PD**	**NRI**	**NTI**
**Mean elevation**	r = 0.15	**r = 0.36**	r = -0.11	r = 0.19	**r = 0.38**	r = -0.12	r = 0.19	r = 0.23	r = -0.1
	p = 0.21	**p < 0.05**	p 0.38	p = 0.1186	**p < 0.05**	p = 0.32	p = 0.13	p = 0.06	p = 0.4
**Slope**	r = 0.05	r = 0.11	r = 0.02	r = 0.05	r = 0.10	r = -0.002	r = 0.04	r = 0.05	r = 0.01
	p = 0.67	p = 0.39	p = 0.88	p = 0.68	p = 0.4	p = 0.99	p = 0.7	p = 0.67	p = 0.9
**Convexity**	**r = 0.33**	**r = 0.44**	r = 0.11	**r = 0.32**	**r = 0.4**	r = 0.1	**r = 0.32**	**r = 0.27**	r = 0.11
	**p = 0.006**	**p < 0.05**	p = 0.38	**p = 0.006**	**p < 0.05**	p = 0.4	**p = 0.006**	**p < 0.05**	p = 0.37

Bold values show significant positive correlations between environmental variables and phylogenetic diversity metrics with respect to different trees (Phylomatic: APG tree, Barcode.con: constrained barcode tree, Barcode.uncon: unconstrained barcode tree) and community data matrices.

## Discussion

### DNA barcode reference database and identification of morphologically undetermined individuals

The first step in DNA barcoding and DNA-based community structure analyses is development of a comprehensive barcode sequence library. In this study, a regional plant *rbcL* and *matK* barcode reference database was successfully generated 3241 individuals reported in the studied subplots of the 25-ha forest research plot. DNA barcode recovery rates were higher for *rbcL* (95.1% of individuals sequenced) than for *matK* (88.5% of individuals sequenced). One reason for this is that the *rbcL* primers worked well across all angiosperms, whereas *matK* is much more difficult to amplify across a wide range of species. However, the use of recently published primer cocktails [70] increased amplification and sequencing of the *matK* barcode compared to earlier studies (e.g. [32]: 69% of species, [33]: 70.4% of species). In total, 69 families were detected by morphology and molecular (barcode) identification. The abundance of these families represents the typical composition of tropical rain forests in Southeast Asia [48, 90, 91]. As expected, the dominant tree family in the examined plots was Dipterocarpaceae with 16% of individuals followed by Euphorbiaceae with 9% (Fig 2). DNA barcodes are especially important when some individuals have not been identified, which is often the case in species-rich tropical forests where it is difficult to obtain flowers and or fruits, which are critical for morphological identification yet often not present when sampling takes place. Using DNA barcodes, phylogenetic trees can be constructed that include morphologically unidentified individuals as long as sequences have been obtained. In addition, families and genera of unidentified individuals can be extracted from BOLD and/or GenBank based on sequence similarity. Here, accessions were successfully assigned to generic or family level using the BOLD Identification System for *rbcL* and *matK* as well as GenBank. However, identification to species level was not achieved because of two issues. Firstly, DNA barcodes, especially *rbcL*, could not distinguish closely related species, leading to more than one high match with the sequences in reference databases. Similar results were observed by Gonzales *et al*. [92] in their study on Amazonian trees, in which neither of the plastid markers tested (including *rbcL* and *matK*), alone or combined, achieved a rate of correct identification greater than 70%. This was especially true for a few species-rich clades that showed little or no variation in these markers. Secondly, some sequences had poor matches in reference databases, reflecting lack of sequences from some species in clades included in our study. Such lack of sequence availability in the reference databases, such as BOLD and GenBank, demonstrates the need for more exhaustive and accurate databases including more species and intra-specific haplotype diversity [93]. Our newly generated sequences make a good contribution to the expansion of these databases.

### Comparison of Phylomatic versus barcode trees

Previous studies have shown that the degree of resolution in community phylogenetic trees plays an important role in detecting non-random patterns of phylogenetic community structure [32, 33, 34, 40, 94]. A high degree of phylogenetic resolution is necessary in phylogenetic community structure analysis because poorly resolved trees can reduce statistical power for detecting non-random forms of community structure, especially when deeper nodes are unresolved [94]. In this study, several approaches were used for phylogenetic reconstruction and compared with respect to resolution and topology: (1) Phylomatic, (2) ML analysis of DNA barcode sequences, and (3) ML analysis with application of a constraint tree (ordinal-level APG topologies). Although time and cost efficient, the Phylomatic approach has disadvantages, for example requiring accurate morphological species identifications at least to family/genus level, because the online phylogenetic query tool requires a list of identified individuals. Furthermore, Phylomatic often provides little or no resolution of phylogenetic relationship among closely related taxa [27]. Compared to the tree obtained by Phylomatic, the barcoding trees yielded better resolution at generic and species levels. An earlier study in a Panamanian forest plot by Kress *et al*. [32] has shown that DNA barcode data alone are sufficient to build phylogenetic trees that closely agree with the APG classification. However, a follow up study in a Puerto Rican forest-dynamics plot showed significantly less concordance with APG [33]. Phylogenetic trees constructed by the use of DNA barcodes often represent single geographic areas, with limited taxon sampling, and therefore lack representatives of many angiosperm families. One could expect such analyses with limited taxon-sampling to differ in topology [95, 96] from the APG classification.

Parallel to the observations in Puerto Rican forest [33], our study showed ordinal-level discrepancies between the tree constructed by Phylomatic and that resulting from ML analysis (Fig 3). In order to build a community tree resolved both at deep and shallow nodes, the ordinal-level APG tree was incorporated as a constraint tree for ML analysis. Many polytomies in the Phylomatic tree were resolved with the barcode data (Fig 4). Proteales were non-monophyletic in the constrained barcode analysis. Although monophyly of the most families was highly supported in our results, three families, Olacaceae, Loganiaceae, and Anacardiaceae (Fig 4), and some genera (e.g. *Shorea*, Dipterocarpaceae; S1 Text) were not monophyletic. This does not mean that the phylogenetic tree reconstructed with the DNA barcodes is wrong, but in many cases reflects non-monophyly of some taxonomic groups (e.g. paraphyly of Olacaceae [97]; and *Shorea* [98]).

### Assessment of phylogenetic community structure and implications

Substantially different results in detection of non-random community structure have been inferred with different phylogenetic approaches (e.g. [32, 34, 40, 94]). For example, in a study of a Chinese subtropical forest, analyses based on a more resolved molecular tree showed more phylogenetic clustering than analyses using a Phylomatic tree [34]. Furthermore, a simulation-based study has shown that measures of phylogenetic diversity and community structure are more sensitive to loss of resolution basally in the tree and less sensitive to loss of resolution terminally [94]. In our study, mean PD was underestimated when calculated based on the Phylomatic tree, which corresponds to Swenson’s observation [94]. Regarding NRI and NTI, different phylogenetic trees gave the same overall result, but the Phylomatic tree detected greater phylogenetic clustering (Fig 6). The well-resolved barcode trees are more likely to influence the inference of patterns of community structure at low taxonomic levels. If competition or interaction with natural pests and diseases is influencing the assembly of co-occurring species, the DNA sequence trees are expected to exhibit lower values of NRI and NTI. The Phylomatic tree not only revealed bias (an upwardly shifted mean in values for NRI and NTI), but also a much greater variance due to noise introduced with the decreased resolution. This corresponds with the results of Kress *et al*. [32], where only two of the five cases of significant phylogenetic structuring detected with analysis using a Phylomatic tree were supported by a barcode tree. On the other hand, analyses based on their barcode tree identified significant phylogenetic structure in five cases for which the Phylomatic approach did not.

In this study, analyses were conducted applying two different community data matrices. To obtain information of the relatedness measures of most individuals, a community data matrix was created based on the assumption that the same morphospecies have identical sequences. This standard approach allows inclusion of individuals for which molecular information is not available. Morphological species identifications in the 25-ha forest dynamics plot are not yet complete, and a large number of species (especially species-rich genera, such as *Aglaia*, *Syzygium*) remain unidentified at the species level (Stuart Davies, personal comm.). However, species-level identification is not an essential aspect of this study, because it is not needed for community structure analysis if DNA sequences are available for a large number of individuals. Therefore, a second analysis was conducted without any morphological identifications, i.e. including only individuals that were sequenced in the community matrix data file. Regardless of which community data matrix was used, having all the individuals from the plot (identified or not at species level) or taking only the sequenced individuals, the general pattern inferred for forest community phylogenetic structure was clustering.

Studies on scale dependence in community phylogenetic analysis of plant communities have shown that phylogenetic clustering increases with spatial scale [47, 99, 100] because this usually includes greater environmental heterogeneity. This leads closely related species sharing environmental factors to sort across contrasting environments [1]. Although there is no standard plot size, we acknowledge that the size of the subplots under investigation (10 × 10 m) is small compared to other studies on phylogenetic community assembly. In our study, six sets of four of the examined 10 × 10 m subplots are adjacent, forming six plots of 20 × 20 m (Fig 1). We compared NRI and NTI of each of the six 20 × 20 m plots with the metrics of the corresponding 10 × 10 m plots and found equivalent results (not shown). Furthermore, as phylogenetic clustering was the general pattern observed in this study, we conclude that the small size of the plots did not negatively bias the detection of phylogenetic clustering.

A central focus in community ecology is the investigation of processes responsible for community assembly, and much research has focused on the phylogenetic consequences of competitive interaction and environmental filtering [101]. We observed phylogenetic clustering in many subplots (S3 Table), contrary to our prediction of phylogenetic overdispersion, which was based upon the dominance of Dipterocarpaceae in Southeast Asian rain forests. Our results of phylogenetic clustering may reflect that Dipterocarpaceae actually account only for 16% (Fig 2) of all trees with ≥ 1 cm diameter in breast height occurring in the studied subplots. Although this is the first study on phylogenetic community assembly in a Southeast Asian forest based on DNA barcode sequences, traditional approaches (i.e. Phylomatic) have been used to explore the phylogenetic structure of tree communities on Indonesian Borneo. Webb [13] found evidence that co-occurring species were more closely related than expected by chance (phylogenetically clustered). Moreover, Webb *et al*. [21], detected overdispersion at seedling level in the same forest, suggesting that sharing of herbivores is important at that life stage but maybe not for adult trees.

Phylogenetic clustering is often used as a proxy for habitat filtering [14]. It has been reported that the floristic composition of mixed dipterocarp forests varies with precipitation, soil nutrients and topography [102, 103]. Moreover, in a study of a species-rich mixed dipterocarp rain forest in Indonesian Borneo, Webb and Peart [104] have shown that distribution and abundance of many species are influenced by local heterogeneity in physical habitat variables. Considering our observations, all three phylogenetic trees revealed significant phylogenetic clustering in most habitats (Fig 7, S3 Table). Furthermore, PD and NRI showed significantly positive correlations with convexity (Table 2), indicating that dynamics of Bruneian forest are, at least partly, shaped by environmental filtering at the community scale. This supports the hypothesis that habitat filtering is an important mechanism responsible for phylogenetic clustering in tropical forests, in accordance with results from most tropical tree communities [13, 35, 41, 47]. On the other hand, those predictions have to be taken with caution because competition might promote phylogenetic clustering [105]. Phylogenetically conserved traits might determine whether a species is a good competitor, which possibly leads to overrepresentation of a clade of good competitors, resulting in phylogenetic clustering [18]. However, further investigations including data on functional niche-associated traits and additional environmental factors (e.g. soil composition) are needed for solid conclusions.

## Conclusion

Although DNA barcodes cannot always be used for species-level identification because reference databases often lack species and haplotype diversity, they can still be useful in species-diverse communities such as tropical rain forests where morphological identification is challenging. In this study, phylogenetic information from two DNA barcoding plastid regions was successfully combined with the APG tree by incorporating ordinal-level constraints on topology. This approach led to a highly resolved tree, which when used in community structure analyses, decreased false positive and false negative observations. The pattern of phylogenetic clustering observed in this study, one of the first using a barcode phylogenetic trees in a Southeast Asian tropical rain forest, gives insights into phylogenetic community structure and corresponds to earlier findings in other tropical forests. Once morphological identification is completed and names of the taxa are validated, the phylogenetic trees constructed here can be used in further studies, and mechanisms responsible for the observed phylogenetic structuring can be identified once niche-associated plant functional traits are integrated.

## Supporting information

S1 Table List of haplotypes included in the study. List of haplotypes, BLAST identifications, BOLD accession numbers, GenBank accession numbers, and vouchers/tree tag numbers.
(DOCX)

S2 Table Mean elevation, slope, and convexity for all subplots. (XLSX)

S3 Table Calculations of PD, MPD, MNTD, NRI, and NTI for each subplot using different trees (Phylomatic, un-, constrained ML trees) and community data matrices. (XLSX)

S1 Text Text file containing different phylogenies reconstructed in this study. (1) the best-scoring ML tree of *rbcL* + *matK* without constraints (2) the best-scoring ML of *rbcL* + *matK* with topological constraints, (3) the dated ultrametric tree (*rbcL* + *matK*) without topological constraints used for community structure analyses, (4) the dated ultrametric tree (*rbcL* + *matk*) with topological constraints used for community structure analyses, (5) the Phylomatic tree, (6) the dated Phylomatic tree used for community structure analyses, (7) the constraint tree. Bootstrap percentages are given for the ML trees 1–2. Abbreviations of names correspond to names in S1 Table.
(NEX)

S2 Text Input file used for dating of the unconstrained ML tree in PATHD8. Abbreviations of names correspond to names in S1 Table.
(TXT)

S3 Text Input file used for dating of the constrained ML tree in PATHD8. Abbreviations of names correspond to names in S1 Table.
(TXT)

S4 Text Community data matrix including sequenced and morphological identified individuals. (TXT)

S5 Text Community data matrix including sequenced individuals only. (TXT)
