# Supplementary material for: Plant DNA barcodes and assessment of phylogenetic community structure of a tropical mixed dipterocarp forest in Brunei Darussalam (Borneo)
Source: PLoS One. 2017 Oct 19;12(10):e0185861. doi: 10.1371/journal.pone.0185861 (PMC5648112; doi:10.1371/journal.pone.0185861)
Supplement: S1 Table — List of haplotypes, BLAST identifications, BOLD accession numbers, GenBank accession numbers, and vouchers/tree tag numbers. (DOCX) [file pone.0185861.s001.docx]

**Dataset S1.**  List of haplotypes (H), BLAST ID, GenBank accession numbers for *rbcL* and *matK*, and voucher/tree tag numbers. ^s^: sequences with stop codons; n/a: sequence not available. Vouchers of the trees are stored under the tree tag number at the Universiti Brunei Darussalam.

| **Haplotype** | **BLAST ID** | **Family/Order** | **BOLD-ID** | ***rbcL*** | ***matK*** | **Vocher/Tree Tag Number** |  |
| --- | --- | --- | --- | --- | --- | --- | --- |
| H1_ILEXCY | *Ilex sp.* | Aquifoliaceae/  Aquifoliales | BABRU2813-15 | MF435396 | MF418661 | 16-2912 |  |
| H2_GONOMI | *Gonocaryum sp.* | Cardiopteraceae/  Aquifoliales | BABRU876-14 | MF435522 | MF418662 | 04-4020 |  |
| H3_GONOSP | *Gonocaryum sp.* | Cardiopteraceae/  Aquifoliales | BABRU2405-15 | MF435521 | n/a | 25-0036 |  |
| H4_GROMQU | *Gomphandra sp.* | Stemonuraceae/  Aquifoliales | BABRU4143-15 | MF435660 | MF418716 | 24-5844 |  |
| H5_STEMMA | Stemonuraceae | Stemonuraceae/  Aquifoliales | BABRU2535-15 | MF435792 | MF418717 | 22-1041 |  |
| H6_STEMUM | Stemonuraceae | Stemonuraceae/  Aquifoliales | BABRU2922-15 | MF435823 | MF418718 | 24-3882 |  |
| H7_PINAAU | Arecaceae | Arecaceae/  Arecales | BABRU1487-15 | MF435835 | MF419145 | 16-2462 |  |
| H8_DRACSP | *Dracaena sp.* | Asparagaceae/  Asparagales | BABRU2080-15 | n/a | MF419146 | 22-0372 |  |
| H9_SALACA | *Salacia sp.* | Celastraceae/  Celastrales | BABRU3098-15 | MF435859 | MF419152 | 24-4063 |  |
| H10_LOPHBE | *Lophopetalum sp.* | Celastraceae/ Celastrales | BABRU1810-15 | MF435870 | MF419153 | 23-3267 |  |
| H11_LOPHSU | *Lophopetalum sp.* | Celastraceae/ Celastrales | BABRU400-14 | MF435291 | MF419154 | 09-4159 |  |
| H12_ALANSP1 | *Alangium sp.* | Cornaceae/ Cornales | BABRU1753-15 | MF43530 | MF418665 | 25-1862 |  |
| H13_ALANJAE | *Alangium sp.* | Cornaceae/ Cornales | BABRU2907-15 | MF435339 | MF418668 | 24-3867 |  |
| H14_ALANJAJ1 | *Alangium sp.* | Cornaceae/ Cornales | BABRU2800-15 | MF435325 | MF418667 | 16-2899 |  |
| H15_ALANJAJ2 | *Alangium sp.* | Cornaceae/ Cornales | BABRU398-14 | MF435314 | MF418666 | 09-4157 |  |
| H16_MASTRO | *Mastixia sp.* | Cornaceae/ Cornales | BABRU3317-15 | MF435351 | MF418664 | 16-4625 |  |
| H17_ANISBE | *Anisophyllea sp.* | Anisophylleaceae/ Cucurbitales | BABRU2252-15 | MF435362 | MF419147 | 24-0498 |  |
| H18_DILLEX | *Dillenia sp.* | Dilleniaceae/ Dilleniales | BABRU3907-15 | MF435373 | MF418758 | 24-5580 |  |
| H19_DILLSU | *Dillenia sp.* | Dilleniaceae/ Dilleniales | BABRU3585-15 | MF435383 | MF418759 | 20-3821 |  |
| H20_SAURSP1 | *Saurauia sp.* | Actinidiaceae/ Ericales | BABRU2382-15 | MF435395 | MF418703 | 25-0013 |  |
| H21_SAURSP2 | *Saurauia sp.* | Actinidiaceae/ Ericales | BABRU1240-14 | MF435407 | MF418706 | 11-4097 |  |
| H22_SAURLO | *Saurauia sp.* | Actinidiaceae/ Ericales | BABRU2487-15 | MF435424 | MF418704 | 23-0210 |  |
| H23_SAURSU | *Saurauia sp.* | Actinidiaceae/ Ericales | BABRU2271-15 | MF435435 | MF418705 | 24-0519 |  |
| H24_DIOSSP2 | *Diospyros sp.* | Ebenaceae/  Ericales | BABRU1449-15 | MF435458 | MF418682 | 13-2673 |  |
| H25_DIOSSP1 | *Diospyros sp.* | Ebenaceae/  Ericales | BABRU3444-15 | MF435446 | MF418679 | 20-3676 |  |
| H26_DIOSSP3 | *Diospyros sp.* | Ebenaceae/ Ericales | BABRU2950-15 | MF435475 | MF418684 | 24-3910 |  |
| H27_DIOSSP4 | *Diospyros sp.* | Ebenaceae/ Ericales | BABRU2222-15 | MF435487 | MF418692 | 16-0891 |  |
| H28_DIOSSP5 | *Diospyros sp.* | Ebenaceae/ Ericales | BABRU2945-15 | MF435486 | n/a | 24-3905 |  |
| H29_DIOSAR | *Diospyros sp.* | Ebenaceae/ Ericales | BABRU473-14 | MF435499 | MF418688 | 11-5698 |  |
| H30_DIOSBO | *Diospyros sp.* | Ebenaceae/ Ericales | BABRU3744-15 | MF435512 | MF418683 | 20-5422 |  |
| H31_DIOSBU | *Diospyros sp.* | Ebenaceae/ Ericales | BABRU1160-14 | MF435520 | MF418695 | 04-4864 |  |
| H32_DIOSCL | *Diospyros sp.* | Ebenaceae/ Ericales | BABRU2221-15 | MF435534 | MF418694 | 16-0890 |  |
| H33_DIOSEL | *Diospyros sp.* | Ebenaceae/ Ericales | BABRU961-14 | MF435550 | MF418696 | 04-4645 |  |
| H34_DIOSFE | *Diospyros sp.* | Ebenaceae/ Ericales | BABRU288-14 | MF435561 | MF418690 | 12-4188 |  |
| H35_DIOKO | *Diospyros sp.* | Ebenaceae/ Ericales | BABRU1963-15 | MF435581 | MF418689 | 16-1786 |  |
| H36_DIOSLA | *Diospyros sp.* | Ebenaceae/ Ericales | BABRU1605-15 | MF435592 | MF418680 | 20-2941 |  |
| H37_DIOSOL | *Diospyros sp.* | Ebenaceae/ Ericales | BABRU3715-15 | MF435606 | MF418681 | 20-5384 |  |
| H38_DIOSPE | *Diospyros sp.* | Ebenaceae/ Ericales | BABRU3371-15 | MF435618 | MF418687 | 20-3602 |  |
| H39_DIOSSB | *Diospyros sp.* | Ebenaceae/ Ericales | BABRU4107-15 | MF435632 | MF418685 | 24-5803 |  |
| H40_DIOSSU | *Diospyros sp.* | Ebenaceae/ Ericales | BABRU1200-14 | MF435648 | MF418693 | 04-4986 |  |
| H41_DIOSTO | *Diospyros sp.* | Ebenaceae/ Ericales | BABRU837-14 | MF435659 | MF418691 | 07-5771 |  |
| H42_DIOSWA | *Diospyros sp.* | Ebenaceae/ Ericales | BABRU2100-15 | MF435672 | MF418686 | 15-0347 |  |
| H43a_BARRCU | *Barringtonia sp.* | Lecythidaceae/ Ericales | BABRU4005-15 | MF435684 | MF418707 | 24-5693 |  |
| H43b_BARRLA | *Barringtonia sp.* | Lecythidaceae/ Ericales | BABRU3337-15 | MF435685 | MF418708 | 16-4645 |  |
| H44_ADINS1 | *Adinandra sp.* | Pentaphylacaceae/ Ericales | BABRU1232-14 | MF435709 | MF418698 | 11-4089 |  |
| H45_ADINAC | *Adinandra sp.* | Pentaphylacaceae/ Ericales | BABRU1856-15 | MF435697 | MF418697 | 13-1859 |  |
| H46_ARDIS1 | *Ardisia sp.* | Primulaceae/ Ericales | BABRU2356-15 | MF435740 | MF418754 | 19-0275 |  |
| H47a_ARDIS3 | *Ardisia sp.* | Primulaceae/ Ericales | BABRU3598-15 | MF435729 | MF418753 | 20-3835 |  |
| H47b_ARDIFE | *Ardisia sp.* | Primulaceae/ Ericales | BABRU4167-15 | MF435728 | MF418752 | 24-5870 |  |
| H48_ARDIS4 | *Ardisia sp.* | Primulaceae/ Ericales | BABRU2227-15 | MF435752 | MF418756 | 16-0896 |  |
| H49_ARDIS2 | *Ardisia sp.* | Primulaceae/ Ericales | BABRU864-14 | MF435768 | MF418755 | 04-4003 |  |
| H50_MADHKI | *Madhuca sp.* | Sapotaceae/ Ericales | BABRU1855-15 | MF435780 | MF418669 | 13-1858 |  |
| H51_PALAS1 | *Palaquium sp.* | Sapotaceae/ Ericales | BABRU2674-15 | MF435817 | MF418677 | 16-2990 |  |
| H52_PALAS2 | *Palaquium sp.* | Sapotaceae/ Ericales | BABRU150-14 | MF435818 | MF418672 | 13-6047 |  |
| H53_PALACA | *Palaquium sp.* | Sapotaceae/ Ericales | BABRU168-14 | MF435791 | MF418674 | 13-6065 |  |
| H54_PALAEL | *Palaquium sp.* | Sapotaceae/ Ericales | BABRU2391-15 | MF435813 | MF418676 | 25-0022 |  |
| H55_PALAGU | *Palaquium sp.* | Sapotaceae/ Ericales | BABRU1846-15 | MF435816 | MF418671 | 13-1848 |  |
| H56_PALASE | *Palaquium sp.* | Sapotaceae/ Ericales | BABRU284-14 | MF435802 | MF418673 | 12-4184 |  |
| H57_SAPOXS1 | *Madhuca sp.* | Sapotaceae/ Ericales | BABRU926-14 | MF435819 | MF418678 | 04-4708 |  |
| H58_SAPOXS2 | *Madhuca sp.* | Sapotaceae/ Ericales | BABRU2693-15 | MF435820 | MF418670 | 16-3065 |  |
| H59_SAPOXS3 | *Manilkara sp.* | Sapotaceae/ Ericales | BABRU4013-15 | MF435821 | MF418675 | 24-5701 |  |
| H60_SYMPSP1 | *Symplocos sp.* | Symplocaceae/ Ericales | BABRU827-14 | MF435822 | MF418699 | 03-5562 |  |
| H61_SYMPSP2 | *Symplocos sp.* | Symplocaceae/ Ericales | BABRU4002-15 | n/a | MF418700 | 24-5690 |  |
| H62_SYMPCO | *Symplocos sp.* | Symplocaceae/ Ericales | BABRU1143-14 | MF435824 | MF418701 | 03-5122 |  |
| H63_SYMPCR | Symplocos sp. | Symplocaceae/ Ericales | BABRU095-14 | MF435825 | MF418702 | 09-3687 |  |
| H64_THEAX | *Tutcheria sp.* | Theaceae/ Fabales | BABRU1462-15 | MF435826 | MF418709 | 16-2436 |  |
| H65_ADENX | *Adenanthera sp.* | Fabaceae/ Fabales | BABRU3185-15 | MF435827 | MF419047 | 16-4488 |  |
| H66_ARCHJI | Fabaceae | Fabaceae/ Fabales | BABRU3537-15 | MF435828 | MF419048 | 20-3772 |  |
| H67_ARCHTR | Fabaceae | Fabaceae/ Fabales | BABRU2545-15 | MF435829 | MF435873 | 22-1051 |  |
| H68_CRUDRE | Fabaceae | Fabaceae/ Fabales | BABRU3714-15 | MF435830 | MF419050 | 20-5383 |  |
| H69_DIALKUKU | *Dialium sp.* | Fabaceae/ Fabales | BABRU1595-15 | MF435832 | n/a | 20-2931 |  |
| H70_DIALKU | *Dialium sp.* | Fabaceae/ Fabales | \|  \| BABRU4228-17 \| \| --- \| --- \| | MF435831 | MF419049 | 24-3849 |  |
| H71_FABABR | *Fordia sp.* | Fabaceae/ Fabales | BABRU2304-15 | MF435693 | MF419064 | 19-0223 |  |
| H72_FORDSP | *Fordia splendidissima* | Fabaceae/ Fabales | BABRU1608-15 | MF435692 | MF419063 | 20-2944 |  |
| H73_KOOMEX^s^ | *Koompassia sp.* | Fabaceae/ Fabales | BABRU2455-15 | MF435833 | MF435890 ^s^ | 25-0349 |  |
| H74_SARADE | *Saraca sp.* | Fabaceae/ Fabales | BABRU794-14 | MF435834 | MF419055 | 01-5515 |  |
| H75a_SINDSP | *Sindora sp.* | Fabaceae/ Fabales | BABRU2734-15 | MF435836 | MF419051 | 16-3107 |  |
| H75b_SINDBE | *Sindora sp.* | Fabaceae/ Fabales | BABRU2188-15 | MF435837 | MF419054 | 16-0856 |  |
| H75c_SINDCO | *Sindora sp.* | Fabaceae/ Fabales | BABRU2403-15 | MF435838 | MF419052 | 25-0034 |  |
| H76_SINDLE | *Sindora sp.* | Fabaceae/ Fabales | BABRU2822-15 | MF435839 | MF419053 | 16-2921 |  |
| H77_XANTSP1^s^ | *Xanthophyllum sp.* | Polygalaceae/ Fabales | BABRU2170-15 | MF435840 | MF435891 *^s^* | 15-0418 |  |
| H78_XANTSP2 | *Xanthophyllum sp.* | Polygalaceae/ Fabales | BABRU1978-15 | MF435841 | MF419056 | 19-1899 |  |
| H79_XANTSP3 | *Xanthophyllum sp.* | Polygalaceae/ Fabales | BABRU4026-15 | n/a | MF419061 | 24-5719 |  |
| H80_XANTBE | *Xanthophyllum sp.* | Polygalaceae/ Fabales | BABRU1192-14 | MF435843 | MF419059^s^ | 04-4951 |  |
| H81_XANTCL | *Xanthophyllum sp.* | Polygalaceae/ Fabales | BABRU1690-15 | MF435844 | MF419058 | 22-1841 |  |
| H82_XANTFE^s^ | *Xanthophyllum sp.* | Polygalaceae/ Fabales | BABRU420-14 | MF435845 | MF435892^s^ | 09-4182 |  |
| H83_XANTGR^s^ | *Xanthophyllum sp.* | Polygalaceae/ Fabales | BABRU1615-15 | MF435847 | MF435893^s^ | 20-2952 |  |
| H84_XANTMA^s^ | *Xanthophyllum sp.* | Polygalaceae/ Fabales | BABRU1923-15 | MF435846 | MF435894^s^ | 16-1741 |  |
| H85_XANTPA | *Xanthophyllum sp.* | Polygalaceae/ Fabales | BABRU3748-15 | MF435848 | MF419057 | 20-5427 |  |
| H86_XANTPE | *Xanthophyllum sp.* | Polygalaceae/ Fabales | BABRU2569-15 | MF435842 | MF419060 | 22-1076 |  |
| H87_XANTRU*^s^* | *Xanthophyllum sp.* | Polygalaceae/ Fabales | BABRU3582-15 | MF435849 | MF435895 | 20-3818 |  |
| H88_XANTST | *Xanthophyllum sp.* | Polygalaceae/ Fabales | BABRU1723-15 | MF435850 | MF419062 | 25-1831 |  |
| H89_CASTSP | *Castanopsis sp.* | Fagaceae/  Fagales | BABRU2352-15 | MF435851 | n/a | 19-0271 |  |
| H90_CASTMO | *Castanopsis sp.* | Fagaceae/  Fagales | BABRU2947-15 | MF435852 | MF419148 | 24-3907 |  |
| H91a_LITHCL | *Lithocarpus sp.* | Fagaceae/  Fagales | BABRU1365-15 | MF435855 | n/a | 13-3482 |  |
| H91b_LITHNI | *Lithocarpus sp.* | Fagaceae/  Fagales | BABRU1307-15 | MF435854 | n/a | 18-3132 |  |
| H92_LITHTE1 | *Lithocarpus sp.* | Fagaceae/  Fagales | BABRU361-14 | MF435856 | MF419150 | 12-5331 |  |
| H93_LITHTE2 | *Lithocarpus sp.* | Fagaceae/  Fagales | BABRU1770-15 | MF435857 | MF419149 | 25-1879 |  |
| H94_LITHLE | *Lithocarpus sp.* | Fagaceae/  Fagales | BABRU1071-14 | MF435853 | MF419151 | 01-3878 |  |
| H95_DYERCO | *Dyera costulata* | Apocynaceae/  Gentianales | BABRU3566-15 | MF435858 | MF418719 | 20-3801 |  |
| H96_TABEAN | *Tabernaemontana sp.* | Apocynaceae/  Gentianales | BABRU077-14 | MF435860 | MF418720 | 09-3667 |  |
| H97_TABEPA | *Tabernaemontana sp.* | Apocynaceae/  Gentianales | BABRU1610-15 | MF435861 | MF418721 | 20-2947 |  |
| H98_NORRMA | *Norrisia sp.* | Loganiaceae/  Gentianales | BABRU1246-14 | MF435862 | MF418757 | 11-4109 |  |
| H99_UTANSP | Loganiaceae | Loganiaceae/  Gentianales | BABRU856-14 | MF435863 | MF419174 | 06-5362 |  |
| H100_RUBIX2 | *Rubiaceae sp.* | Rubiaceae/  Gentianales | BABRU898-14 | MF435283 | MF418728 | 04-4078 |  |
| H101_RUBIX1 | *Rubiaceae sp.* | Rubiaceae/  Gentianales | BABRU2058-15 | MF435864 | MF418722 | 22-0348 |  |
| H102_AIDISP1 | *Rubiaceae sp.* | Rubiaceae/  Gentianales | BABRU881-14 | MF435865 | MF418723 | 04-4027 |  |
| H103_AIDISP2 | *Rubiaceae sp.* | Rubiaceae/ Gentianales | BABRU1395-15 | MF435866 | MF418724 | 13-3513 |  |
| H104_CANTCO | *Rubiaceae sp.* | Rubiaceae/ Gentianales | BABRU079-14 | MF435867 | MF418730 | 09-3669 |  |
| H105_DISCSP | *Rubiaceae sp.* | Rubiaceae/ Gentianales | BABRU1095-14 | MF435868 | MF418725 | 01-3921 |  |
| H106_GAERVA | *Gaertnera sp.* | Rubiaceae/ Gentianales | BABRU2334-15 | MF435869 | MF418749 | 19-0253 |  |
| H107_GARDEL | *Gardenia sp.* | Rubiaceae/ Gentianales | BABRU2143-15 | MF435281 | MF418726 | 15-0391 |  |
| H108_GARDLO | *Gardeniopsis longifolia* | Rubiaceae/  Gentianales | BABRU3392-15 | MF435282 | MF418743 | 20-3623 |  |
| H109_IXORSP3 | *Ixora sp.* | Rubiaceae/  Gentianales | BABRU2433-15 | MF435288 | MF418735 | 25-0326 |  |
| H110_IXORSP1 | *Ixora sp.* | Rubiaceae/  Gentianales | BABRU1562-15 | MF435284 | MF418731 | 20-2897 |  |
| H111_IXORSP2 | *Ixora sp.* | Rubiaceae/  Gentianales | BABRU085-14 | MF435285 | MF418734 | 09-3675 |  |
| H112_IXORGL | *Ixora sp.* | Rubiaceae/  Gentianales | BABRU1072-14 | MF435286 | MF418733 | 01-3879 |  |
| H113_IXORPU | *Ixora sp.* | Rubiaceae/  Gentianales | BABRU090-14 | MF435287 | MF418732 | 09-3681 |  |
| H114_LASIRO | *Lasianthus sp.* | Rubiaceae/  Gentianales | BABRU4076-15 | MF435810 | MF435877 | 24-5772 |  |
| H115_NEONSP | *Neonauclea sp.* | Rubiaceae/  Gentianales | BABRU1229-14 | MF435289 | MF418736 | 11-4085 |  |
| H116_NEONSU | *Neonauclea sp.* | Rubiaceae/  Gentianales | BABRU3578-15 | MF435290 | MF418737 | 20-3814 |  |
| H117_PLEISP | Rubiaceae | Rubiaceae/ Gentianales | BABRU1265-14 | MF435811 | MF435878 | 11-4132 |  |
| H118_PARVSP | Rubiaceae | Rubiaceae/ Gentianales | BABRU3756-15 | MF435292 | MF418738 | 20-5440 |  |
| H119_PORTCA | *Porterandia sp.* | Rubiaceae/ Gentianales | BABRU221-14 | MF435293 | MF418727 | 14-4342 |  |
| H120a_PRISSP | *Prismatomeris sp.* | Rubiaceae/ Gentianales | BABRU4200-15 | MF435294 | MF418744 | 24-5905 |  |
| H120b_PRISBE | *Prismatomeris sp.* | Rubiaceae/ Gentianales | BABRU4198-15 | MF435295 | MF418745 | 24-5903 |  |
| H121_PSYCSP | *Psychotria sp.* | Rubiaceae/ Gentianales | BABRU3315-15 | MF435296 | MF418747 | 16-4623 |  |
| H122_RENNEL | Rubiaceae | Rubiaceae/ Gentianales | BABRU3579-15 | MF435297 | MF418746 | 20-3815 |  |
| H123_SAPRSP | *Saprosma sp.* | Rubiaceae/ Gentianales | BABRU1494-15 | MF435298 | MF418748 | 16-2471 |  |
| H124_TARESP1 | *Tarenna sp.* | Rubiaceae/ Gentianales | BABRU1589-15 | MF435299 | MF418729 | 20-2925 |  |
| H125_TARESP2 | *Tarenna sp.* | Rubiaceae/ Gentianales | BABRU3913-15 | MF435300 | n/a | 24-5586 |  |
| H126_UROPS2 | *Urophyllum sp.* | Rubiaceae/ Gentianales | BABRU4125-15 | MF435304 | MF418742 | 24-5821 |  |
| H127_UROPS1 | *Urophyllum sp.* | Rubiaceae/ Gentianales | BABRU4097-15 | MF435302 | MF418741 | 24-5793 |  |
| H128_UROPSP3 | *Urophyllum sp.* | Rubiaceae/ Gentianales | BABRU2572-15 | MF435305 | MF418740 | 22-1079 |  |
| H129_UROPSP4 | *Urophyllum sp.* | Rubiaceae/ Gentianales | BABRU838-14 | MF435306 | n/a | 07-5773 |  |
| H130_UROPSP5 | *Urophyllum sp.* | Rubiaceae/ Gentianales | BABRU2810-15 | MF435307 | n/a | 16-2909 |  |
| H131_UROPCO | *Urophyllum sp.* | Rubiaceae/ Gentianales | BABRU202-14 | MF435301 | MF418739 | 14-4323 |  |
| H132_UROPWO | *Urophyllum sp.* | Rubiaceae/ Gentianales | BABRU4041-15 | MF435308 | n/a | 24-5736 |  |
| H133_GNETGN | *Gnetum sp.* | Gnetaceae/ Gnetales | BABRU2567-15 | MF435309 | n/a | 22-1074 |  |
| H134_TEIJCO | Lamiaceae | Lamiaceae/ Lamiales | BABRU440-14 | MF435310 | MF418711 | 09-4203 |  |
| H135_TEIJSI | Lamiaceae | Lamiaceae/ Lamiales | BABRU1683-15 | MF435311 | MF418712 | 22-1834 |  |
| H136_VITEVI | *Vitex sp.* | Lamiaceae/ Lamiales | BABRU1930-15 | MF435312 | MF418713 | 16-1749 |  |
| H137_CHIOSP4 | *Oleaceae sp.* | Oleaceae/ Lamiales | BABRU2331-15 | MF435313 | MF418714 | 19-0250 |  |
| H138_OLEASP | *Oleaceae sp.* | Oleaceae/ Lamiales | BABRU2992-15 | MF435315 | MF418715 | 24-3952 |  |
| H139_ALSEBA | Lauracee | Lauraceae/ Laurales | BABRU3775-15 | MF435319 | MF419083 | 20-5461 |  |
| H140_ACTISP | *Actinodaphne sp.* | Lauraceae/ Laurales | BABRU2765-15 | MF435318 | MF419066 | 16-3019 |  |
| H141_ACTIBO | *Actinodaphne sp* | Lauraceae/ Laurales | BABRU2024-15 | MF435316 | MF419065 | 19-1945 |  |
| H142_ACTIPR | *Actinodaphne sp* | Lauraceae/ Laurales | BABRU3546-15 | MF435317 | MF419082 | 20-3781 |  |
| H143_BEILGL | *Beilschmiedia sp.* | Lauraceae/ Laurales | BABRU1036-14 | MF435321 | n/a | 01-3840 |  |
| H144_BEILTA | *Beilschmiedia sp.* | Lauraceae/ Laurales | BABRU888-14 | MF435320 | MF419086 | 04-4061 |  |
| H145_CINNJA | Lauraceae | Lauraceae/ Laurales | BABRU3728-15 | MF435322 | MF419081 | 20-5404 |  |
| H146_CRYPER | *Cryptocarya sp.* | Lauraceae/ Laurales | BABRU1967-15 | MF435324 | n/a | 16-1790 |  |
| H147_CRYPTO | *Cryptocarya sp.* | Lauraceae/ Laurales | BABRU356-14 | MF435323 | MF419088 | 12-5325 |  |
| H148_ENDICO | *Endiandra sp.* | Lauraceae/ Laurales | BABRU3845-15 | MF435326 | MF419087 | 20-5554 |  |
| H149_LITSSP6 | *Litsea sp.* | Lauraceae/ Laurales | BABRU3299-15 | MF435331 | n/a | 16-4606 |  |
| H150a_LITSSP9 | *Litsea sp.* | Lauraceae/ Laurales | BABRU3161-15 | MF435334 | MF419070 | 16-4462 |  |
| H150b_LITSSP10 | *Litsea sp.* | Lauraceae/ Laurales | BABRU1824-15 | MF435335 | MF419076 | 23-3282 |  |
| H150c_LITSFU | *Litsea sp.* | Lauraceae/ Laurales | BABRU1322-15 | MF435336 | MF419071 | 18-3149 |  |
| H150d_LITSLA | *Litsea sp.* | Lauraceae/ Laurales | BABRU1510-15 | MF435337 | MF419072 | 16-2488 |  |
| H151_LITSSP8 | *Litsea sp.* | Lauraceae/ Laurales | BABRU2007-15 | MF435333 | MF419069 | 19-1928 |  |
| H152_LITSSP7 | *Litsea sp.* | Lauraceae/ Laurales | BABRU1926-15 | MF435332 | MF419085 | 16-1745 |  |
| H153_LITSAC1 | *Litsea sp.* | Lauraceae/ Laurales | BABRU1188-14 | MF435340 | MF419077 | 04-4944 |  |
| H154_LITSAC2 | *Litsea sp.* | Lauraceae/ Laurales | BABRU2353-15 | MF435341 | MF419079 | 19-0272 |  |
| H155_LITSCA | *Litsea sp.* | Lauraceae/ Laurales | BABRU2615-15 | MF435342 | MF419074 | 22-1125 |  |
| H156_LITSCO | *Litsea sp.* | Lauraceae/ Laurales | BABRU1839-15 | MF435343 | MF419075 | 13-1841 |  |
| H157_LITSFE | *Litsea sp.* | Lauraceae/ Laurales | BABRU4004-15 | MF435344 | MF435874 | 24-5692 |  |
| H158a_LITSME | *Litsea sp.* | Lauraceae/ Laurales | BABRU3842-15 | MF435812 | MF435880 | 20-5551 |  |
| H158b_LITSAN | *Litsea sp.* | Lauraceae/ Laurales | BABRU371-14 | MF435338 | MF435879 | 08-5660 |  |
| H159_LITSRU | *Litsea rubicunda* | Lauraceae/ Laurales | BABRU4161-15 | MF435345 | MF419080 | 24-5864 |  |
| H160_LITSSP1 | *Litsea sp.* | Lauraceae/ Laurales | BABRU3859-15 | MF435327 | MF419067 | 20-5568 |  |
| H161_LITSSP2 | *Litsea sp.* | Lauraceae/ Laurales | BABRU3472-15 | MF435328 | MF419073 | 20-3704 |  |
| H162_LITSSP3 | *Litsea sp.* | Lauraceae/ Laurales | BABRU3722-15 | MF435329 | MF419068 | 20-5393 |  |
| H163_LITSSP4 | *Litsea sp.* | Lauraceae/ Laurales | BABRU3944-15 | n/a | MF419078 | 24-5621 |  |
| H164_LITSSP5 | *Litsea sp.* | Lauraceae/ Laurales | BABRU941-14 | MF435330 | n/a | 04-4619 |  |
| H165_PHOEGR | Lauraceae | Lauraceae/ Laurales | BABRU457-14 | MF435346 | MF419084 | 08-6445 |  |
| H167_PROTME | *Potoxylon melagangai* | Lauraceae/ Laurales | BABRU830-14 | MF435347 | MF419089 | 03-5567 |  |
| H168_ANNOX1 | *Annonaceae sp.* | Annonaceae/ Magnoliales | BABRU820-14 | MF435348 | MF419093 | 01-5545 |  |
| H169_ANNOX2 | *Annonaceae sp.* | Annonaceae/ Magnoliales | BABRU824-14 | MF435349 | MF419117 | 01-5549 |  |
| H170_ANNOX3 | *Annonaceae sp.* | Annonaceae/ Magnoliales | BABRU805-14 | MF435350 | MF419094 | 01-5526 |  |
| H171_ANNOX4 | *Annonaceae sp.* | Annonaceae/ Magnoliales | BABRU3017-15 | MF435352 | MF419097 | 24-3979 |  |
| H172_ANNOX5 | Annonaceae | Annonaceae/ Magnoliales | BABRU1913-15 | MF435388 | MF419101 | 25-2695 |  |
| H173_ANNOX6 | Annonaceae | Annonaceae/ Magnoliales | BABRU064-14 | MF435389 | MF419102 | 09-3654 |  |
| H174_XYLOMA | *Xylopia sp.* | Annonaceae/ Magnoliales | BABRU2532-15 | MF435387 | MF419123 | 13-0920 |  |
| H175_DREPHA | Annonaceae | Annonaceae/ Magnoliales | BABRU214-14 | MF435353 | MF419121 | 14-4335 |  |
| H176_GONISP | *Goniothalamus sp.* | Annonaceae/ Magnoliales | BABRU309-14 | MF435356 | MF419125 | 12-5725 |  |
| H177_GONIME | *Goniothalamus sp.* | Annonaceae/ Magnoliales | BABRU2627-15 | MF435354 | MF419124 | 22-1137 |  |
| H178_GONIPA | *Goniothalamus sp.* | Annonaceae/ Magnoliales | BABRU2204-15 | MF435355 | MF419126 | 16-0872 |  |
| H179_GONITA | *Goniothalamus sp.* | Annonaceae/ Magnoliales | BABRU025-14 | MF435357 | MF419128 | 05-5376 |  |
| H180_GONIVE | *Goniothalamus sp.* | Annonaceae/ Magnoliales | BABRU2543-15 | MF435358 | MF419127 | 22-1049 |  |
| H181_HUBERU | Annonaceae | Annonaceae/ Magnoliales | BABRU3448-15 | MF435359 | MF419118 | 20-3680 |  |
| H182_MAASGL | *Maasia sp.* | Annonaceae/ Magnoliales | BABRU172-14 | MF435360 | MF419120 | 13-6069 |  |
| H183_MAASSU | *Maasia sp.* | Annonaceae/ Magnoliales | BABRU1334-15 | MF435361 | MF419119 | 13-3449 |  |
| H184_MEIOSP | Annonaceae | Annonaceae/ Magnoliales | BABRU2417-15 | MF435363 | MF419099 | 25-0309 |  |
| H185_MEZZMA | *Mezzettia sp.* | Annonaceae/ Magnoliales | BABRU1381-15 | MF435364 | MF419122 | 13-3498 |  |
| H186_MITRLO | Annonaceae | Annonaceae/ Magnoliales | BABRU3840-15 | MF435365 | MF419106 | 20-5549 |  |
| H187_MITRMA | Annonaceae | Annonaceae/ Magnoliales | BABRU1106-14 | MF435366 | MF419100 | 03-5083 |  |
| H188_MONOEU | *Monocarpia sp.* | Annonaceae/ Magnoliales | BABRU2441-15 | MF435367 | MF419105 | 25-0335 |  |
| H189_MONOLA | Annonaceae | Annonaceae/ Magnoliales | BABRU4169-15 | MF435368 | MF419096 | 24-5872 |  |
| H190_NEOUPR | *Neo-uvaria sp.* | Annonaceae/ Magnoliales | BABRU3822-15 | MF435369 | MF419098 | 20-5521 |  |
| H191_PHAESP | Annonaceae | Annonaceae/ Magnoliales | BABRU3480-15 | MF435370 | MF419095 | 20-3712 |  |
| H192_PHAEOP | *Phaeanthus sp.* | Annonaceae/ Magnoliales | BABRU2265-15 | MF435371 | MF419116 | 24-0512 |  |
| H193_POLYSP1 | *Polyalthia sp.* | Annonaceae/ Magnoliales | BABRU3347-15 | MF435372 | MF419109 | 16-4655 |  |
| H194_POLYSP3 | *Polyalthia sp.* | Annonaceae/ Magnoliales | BABRU4148-15 | MF435378 | MF419115 | 24-5851 |  |
| H195_POLYSP5 | *Polyalthia sp.* | Annonaceae/ Magnoliales | BABRU3397-15 | MF435379 | MF419112 | 20-3628 |  |
| H196_POLYBO | *Polyalthia sp.* | Annonaceae/ Magnoliales | BABRU840-14 | MF435374 | MF419111 | 06-5345 |  |
| H197_POLYBU | *Polyalthia sp.* | Annonaceae/ Magnoliales | BABRU1065-14 | MF435375 | MF419110 | 01-3871 |  |
| H198_POLYCA1 | *Polyalthia sp.* | Annonaceae/ Magnoliales | BABRU2220-15 | MF435376 | MF419114 | 16-0889 |  |
| H199_POLYCA2 | *Polyalthia sp.* | Annonaceae/ Magnoliales | BABRU148-14 | MF435377 | MF419113 | 13-6045 |  |
| H200a_POPOHI | *Popowia* | Annonaceae/ Magnoliales | BABRU1800-15 | MF435381 | MF419107 | 23-3256 |  |
| H200b_POPOPI | *Popowia* | Annonaceae/ Magnoliales | BABRU1830-15 | MF435382 | MF419108 | 23-3288 |  |
| H201_POPOSP1 | *Popowia sp.* | Annonaceae/ Magnoliales | BABRU2654-15 | MF435380 | MF435875 | 16-2970 |  |
| H202_SAGELA | *Sageraea* | Annonaceae/ Magnoliales | BABRU3419-15 | MF435384 | MF419103 | 20-3650 |  |
| H203_SAGESA | *Sageraea* | Annonaceae/ Magnoliales | BABRU2628-15 | MF435385 | MF419104 | 22-1138 |  |
| H204_UVARSXI | *Uvaria sp.* | Annonaceae/ Magnoliales | BABRU313-14 | MF435386 | MF419129 | 12-5280 |  |
| H205_MAGNS1 | *Magnolia sp.* | Magnoliaceae/ Magnoliales | BABRU432-14 | MF435390 | MF419090 | 09-4194 |  |
| H206_MAGNAS | *Magnolia sp.* | Magnoliaceae/ Magnoliales | BABRU424-14 | MF435391 | MF419092 | 09-4186 |  |
| H207_MAGNGI | *Magnolia sp.* | Magnoliaceae/ Magnoliales | BABRU345-14 | MF435392 | MF419091 | 12-5313 |  |
| H208_GYMNFA | *Gymnacranthera sp.* | Myristicaceae/ Magnoliales | BABRU4084-15 | MF435400 | n/a | 24-5780 |  |
| H209_GYMNFO | *Gymnacranthera sp.* | Myristicaceae/ Magnoliales | BABRU4205-15 | MF435401 | MF419131 | 24-5910 |  |
| H210_HORSGR | *Horsfieldia sp.* | Myristicaceae/ Magnoliales | BABRU239-14 | MF435402 | MF419142 | 12-4139 |  |
| H211_HORSFL | *Horsfieldia sp.* | Myristicaceae/ Magnoliales | BABRU3696-15 | MF435403 | MF419143 | 20-5358 |  |
| H212_KNEMS1 | *Knema sp.* | Myristicaceae/ Magnoliales | BABRU2187-15 | MF435404 | MF419134 | 16-0855 |  |
| H213_KNEMS2 | *Knema sp.* | Myristicaceae/ Magnoliales | BABRU3072-15 | MF435405 | MF419138 | 24-4037 |  |
| H214_KNEMS3 | *Knema sp.* | Myristicaceae/ Magnoliales | BABRU3360-15 | MF435406 | MF419135 | 16-4669 |  |
| H215_KNEMS6 | *Knema sp.* | Myristicaceae/ Magnoliales | BABRU949-14 | MF435410 | n/a | 04-4633 |  |
| H216_KNEMS7 | *Knema sp.* | Myristicaceae/ Magnoliales | BABRU819-14 | MF435399 | MF419133 | 01-5544 |  |
| H217_KNEMS4 | *Knema sp.* | Myristicaceae/ Magnoliales | BABRU2516-15 | MF435408 | MF419136 | 13-0899 |  |
| H218_KNEMS5 | *Knema sp.* | Myristicaceae/ Magnoliales | BABRU4191-15 | MF435409 | MF419141 | 24-5896 |  |
| H219_KNEMSE | *Knema sp.* | Myristicaceae/ Magnoliales | BABRU842-14 | MF435411 | MF419137 | 06-5347 |  |
| H220_MYRXS1 | *Myristicaceae sp.* | Myristicaceae/ Magnoliales | BABRU1672-15 | MF435393 | MF419130 | 22-1821 |  |
| H221_MYRXS2 | *Myristicaceae sp.* | Myristicaceae/ Magnoliales | BABRU2228-15 | MF435394 | MF419132 | 16-0898 |  |
| H222_MYRXSIII | *Myristica sp.* | Myristicaceae/ Magnoliales | BABRU155-14 | MF435398 | MF419140 | 13-6052 |  |
| H223_MYRIS4 | *Myristica sp.* | Myristicaceae/ Magnoliales | BABRU3697-15 | MF435397 | MF419139 | 20-5360 |  |
| H224_HYDNS1 | *Hydnocarpus sp.* | Achariaceae/ Malpighiales | BABRU4109-15 | MF435412 | MF418945 | 24-5805 |  |
| H225a_HYDNS2 | *Hydnocarpus sp.* | Achariaceae/ Malpighiales | BABRU872-14 | MF435414 | MF435881 | 04-4011 |  |
| H225b_HYDNBO | *Hydnocarpus sp.* | Achariaceae/ Malpighiales | BABRU3110-15 | MF435415 | MF418949 | 24-4075 |  |
| H225c_HYDNSB | *Hydnocarpus sp.* | Achariaceae/ Malpighiales | BABRU2726-15 | MF435416 | MF418950 | 16-3099 |  |
| H225d_HYDNSM | *Hydnocarpus sp.* | Achariaceae/ Malpighiales | BABRU1722-15 | MF435417 | MF418951 | 22-1874 |  |
| H226_HYDNKU | *Hydnocarpus sp.* | Achariaceae/ Malpighiales | BABRU3189-15 | MF435413 | MF418946 | 16-4492 |  |
| H227_HYDNPI | *Hydnocarpus sp.* | Achariaceae/ Malpighiales | BABRU3119-15 | MF435418 | MF418948 | 16-4419 |  |
| H228_HYDNWO | *Hydnocarpus sp.* | Achariaceae/ Malpighiales | BABRU896-14 | MF435419 | MF418947 | 04-4070 |  |
| H229a_RYPAS1 | Achariaceae | Achariaceae/ Malpighiales | BABRU1018-14 | MF435420 | MF418952 | 01-3822 |  |
| H229b_RYPAQU | Achariaceae | Achariaceae/ Malpighiales | BABRU1498-15 | MF435421 | MF418954 | 16-2475 |  |
| H229c_RYPAHU | Achariaceae | Achariaceae/ Malpighiales | BABRU125-14 | MF435422 | MF418953 | 10-5215 |  |
| H230d_RYPAHI | Achariaceae | Achariaceae/ Malpighiales | BABRU427-14 | MF435423 | MF435882 | 09-4189 |  |
| H231_CALLS3 | *Calophyllum sp.* | Callophyllaceae/ Malpighiales | BABRU2431-15 | MF435427 | MF419157 | 25-0324 |  |
| H232_CALLS1 | *Calophyllum sp.* | Callophyllaceae/ Malpighiales | BABRU2574-15 | MF435425 | MF419155 | 22-1081 |  |
| H233_CALLS2 | *Calophyllum sp.* | Callophyllaceae/ Malpighiales | BABRU807-14 | MF435426 | MF419158 | 01-5528 |  |
| H234_CALLDE | *Calophyllum sp.* | Callophyllaceae/ Malpighiales | BABRU2691-15 | MF435428 | MF419160 | 16-3063 |  |
| H235_CALLFE | *Calophyllum sp.* | Callophyllaceae/ Malpighiales | BABRU2378-15 | MF435429 | MF419156 | 25-0009 |  |
| H236_CALLWO | *Calophyllum sp.* | Callophyllaceae/ Malpighiales | BABRU2930-15 | MF435430 | MF419159 | 24-3890 |  |
| H237_KAYES1 | *Kayea sp.* | Callophyllaceae/ Malpighiales | BABRU3178-15 | MF435431 | MF419161 | 16-4479 |  |
| H238_KAYEFE | *Kayea sp.* | Callophyllaceae/ Malpighiales | BABRU2633-15 | MF435432 | MF419162 | 16-2949 |  |
| H239_KAYEMA | *Kayea sp.* | Callophyllaceae/ Malpighiales | BABRU3420-15 | MF435433 | MF419163 | 20-3651 |  |
| H240_KAYEOB | *Kayea sp.* | Callophyllaceae/ Malpighiales | BABRU3824-15 | MF435434 | MF419164 | 20-5527 |  |
| H241_BHESPA | *Bhesa paniculata* | Centroplacaceae/ Malpighiales | BABRU4163-15 | MF435436 | MF419020 | 24-5866 |  |
| H242_ATUNRA | *Atuna sp.* | Chrysobalanaceae/ Malpighiales | BABRU1050-14 | MF435803 | MF435883 | 01-3854 |  |
| H243_PARIEL | *Parinari sp.* | Chrysobalanaceae/ Malpighiales | BABRU1225-14 | MF435805 | MF435884 | 04-5020 |  |
| H244_KOSTHE | *Kostermanthus sp.* | Chrysobalanaceae/ Malpighiales | BABRU3399-15 | MF435814 | n/a | 20-3630 |  |
| H245_GARCSPI | *Garcinia sp.* | Clusiaceae/ Malpighiales | BABRU2795-15 | MF435437 | MF419166 | 16-2894 |  |
| H246_GARCSP2 | *Garcinia sp.* | Clusiaceae/ Malpighiales | BABRU1517-15 | MF435438 | MF419172 | 20-2851 |  |
| H247_GARCS7 | *Garcinia sp.* | Clusiaceae/ Malpighiales | BABRU1968-15 | MF435443 | MF419170 | 19-1887 |  |
| H248_GARCS4 | *Garcinia sp.* | Clusiaceae/ Malpighiales | BABRU1114-14 | MF435440 | MF419167 | 03-5091 |  |
| H249_GARCS5 | *Garcinia sp.* | Clusiaceae/ Malpighiales | BABRU2181-15 | MF435441 | MF435876 | 15-0431 |  |
| H250_GARCS6 | *Garcinia sp.* | Clusiaceae/ Malpighiales | BABRU2814-15 | MF435442 | MF419168 | 16-2913 |  |
| H251_GARCSP3 | *Garcinia sp.* | Clusiaceae/ Malpighiales | BABRU3492-15 | MF435439 | MF419169 | 20-3725 |  |
| H252_GARCHA | *Garcinia sp.* | Clusiaceae/ Malpighiales | BABRU308-14 | MF435444 | MF419173 | 12-5274 |  |
| H253_GARCLA | *Garcinia sp.* | Clusiaceae/ Malpighiales | BABRU1569-15 | MF435445 | n/a | 20-2904 |  |
| H254_GARCMA | *Garcinia sp.* | Clusiaceae/ Malpighiales | BABRU2236-15 | MF435808 | n/a | 16-0908 |  |
| H255_GARCSA | *Garcinia sp.* | Clusiaceae/ Malpighiales | BABRU1565-15 | MF435447 | MF419171 | 20-2900 |  |
| H256_CTENPA | *Ctenolophon sp.* | Ctenolophonaceae/ Malpighiales | BABRU1463-15 | MF435448 | MF418992 | 16-2437 |  |
| H257a_BLUMBO | *Blumeodendron sp.* | Euphorbiaceae/ Malpighiales | BABRU2138-15 | MF435449 | MF418958 | 15-0385 |  |
| H257b_BLUMCA | *Blumeodendron sp.* | Euphorbiaceae/ Malpighiales | BABRU2474-15 | MF435450 | MF418959 | 23-0184 |  |
| H258_BLUMKU | *Blumeodendron sp.* | Euphorbiaceae/ Malpighiales | BABRU2194-15 | MF435451 | MF418961 | 16-0862 |  |
| H259_BLUMSU | *Blumeodendron sp.* | Euphorbiaceae/ Malpighiales | BABRU3523-15 | MF435452 | MF418960 | 20-3757 |  |
| H260_BLUMTO | *Blumeodendron sp.* | Euphorbiaceae/ Malpighiales | BABRU1404-15 | MF435453 | MF418962 | 13-3522 |  |
| H261_CEPHMA | *Cephalomappa sp.* | Euphorbiaceae/ Malpighiales | BABRU2458-15 | MF435815 | MF418982 | 25-0352 |  |
| H262_CHAECA | *Chaetocarpus castanocarpus* | Euphorbiaceae/ Malpighiales | BABRU2929-15 | MF435454 | MF418984 | 24-3889 |  |
| H263_CHEIMO | *Cheilosa montana* | Euphorbiaceae/ Malpighiales | BABRU1055-14 | MF435455 | MF418955 | 01-3859 |  |
| H264_ELATTA | *Elateriospermum tapos* | Euphorbiaceae/ Malpighiales | BABRU2013-15 | MF435456 | MF418977 | 19-1934 |  |
| H265_ERISLE | *Erismanthus leembruggianus* | Euphorbiaceae/ Malpighiales | BABRU1123-14 | MF435457 | MF418978 | 03-5100 |  |
| H266_HANCEU | *Hancea eucaustus* | Euphorbiaceae/ Malpighiales | BABRU408-14 | MF435459 | MF418976 | 09-4167 |  |
| H267_KOILLO | *Koilodepas sp.* | Euphorbiaceae/ Malpighiales | BABRU453-14 | MF435460 | MF418983 | 08-6441 |  |
| H268a_MACASP | *Macaranga sp.* | Euphorbiaceae/ Malpighiales | BABRU3916-15 | MF435461 | MF418964 | 24-5589 |  |
| H268b_MACAHU | *Macaranga sp.* | Euphorbiaceae/ Malpighiales | BABRU3699-15 | MF435462 | MF418965 | 20-5363 |  |
| H268c_MACALA | *Macaranga sp.* | Euphorbiaceae/ Malpighiales | BABRU2771-15 | MF435463 | MF418966 | 16-3025 |  |
| H268_MACATR | *Macaranga sp.* | Euphorbiaceae/ Malpighiales | BABRU1864-15 | MF435464 | MF418967 | 13-1868 |  |
| H269_MACABE | *Macaranga sp.* | Euphorbiaceae/ Malpighiales | BABRU1917-15 | MF435465 | MF418968 | 25-2701 |  |
| H270a_MACABR | *Macaranga sp.* | Euphorbiaceae/ Malpighiales | BABRU892-14 | MF435466 | MF418969 | 04-4066 |  |
| H270b_MACAHO | *Macaranga sp.* | Euphorbiaceae/ Malpighiales | BABRU3431-15 | MF435467 | MF418971 | 20-3663 |  |
| H270c_MACALO | *Macaranga sp.* | Euphorbiaceae/ Malpighiales | BABRU3837-15 | MF435468 | MF418970 | 20-5546 |  |
| H271_MALLS1 | *Mallotus sp.* | Euphorbiaceae/ Malpighiales | BABRU2241-15 | MF435469 | MF418972 | 16-0914 |  |
| H272_MALLS2 | *Mallotus sp.* | Euphorbiaceae/ Malpighiales | BABRU1651-15 | MF435470 | MF418974 | 22-2146 |  |
| H273_MALLLE | *Mallotus sp.* | Euphorbiaceae/ Malpighiales | BABRU1166-14 | MF435471 | MF418975 | 04-4870 |  |
| H274_MALLWR | *Mallotus sp.* | Euphorbiaceae/ Malpighiales | BABRU431-14 | MF435472 | MF418973 | 09-4193 |  |
| H275_NEOSKI | *Neoscortechinia sp.* | Euphorbiaceae/ Malpighiales | BABRU1885-15 | MF435473 | MF418956 | 13-1890 |  |
| H276_NEOSSU | *Neoscortechinia sp.* | Euphorbiaceae/ Malpighiales | BABRU3411-15 | MF435474 | MF418957 | 20-3642 |  |
| H277_PIMEGR1 | *Pimelodendron sp.* | Euphorbiaceae/ Malpighiales | BABRU3569-15 | MF435476 | MF418979 | 20-3804 |  |
| H278_PIMEGR2 | *Pimelodendron sp.* | Euphorbiaceae/ Malpighiales | BABRU141-14 | MF435477 | MF418980 | 10-5232 |  |
| H279_PIMEGR3 | *Pimelodendron sp.* | Euphorbiaceae/ Malpighiales | BABRU415-14 | MF435478 | MF418981 | 09-4177 |  |
| H280_PTYCAR | Euphorbiaceae | Euphorbiaceae/ Malpighiales | BABRU2057-15 | MF435479 | MF418963 | 22-0347 |  |
| H281_TRIGMA | *Trigonopleura malayana* | Euphorbiaceae/ Malpighiales | BABRU3275-15 | MF435480 | MF418985 | 16-4582 |  |
| H282_CRATFO | *Cratoxylum sp.* | Hypericaceae/ Malpighiales | BABRU3368-15 | MF435481 | MF419165 | 16-4679 |  |
| H283_GALEFU | *Galearia sp.* | Pandaceae/ Malpighiales | BABRU3706-15 | MF435482 | MF418986 | 20-5373 |  |
| H284_CARRSP | *Carralia sp.* | Rhizophoraceae/ Malpighiales | BABRU1425-15 | MF435483 | MF419002 | 13-2647 |  |
| H285_ANTISP1 | *Antidesma sp.* | Rhizophoraceae/ Malpighiales | BABRU920-14 | MF435484 | n/a | 04-4494 |  |
| H286_ANTILI | *Antidesma sp.* | Rhizophoraceae/ Malpighiales | BABRU1593-15 | MF435489 | MF419005 | 20-2929 |  |
| H287_ANTISP2 | *Antidesma sp.* | Rhizophoraceae/ Malpighiales | BABRU379-14 | MF435485 | MF419003 | 08-5671 |  |
| H288_ANTILE | *Antidesma sp.* | Rhizophoraceae/ Malpighiales | BABRU446-14 | MF435488 | MF419004 | 08-6434 |  |
| H289_ANTIST | *Antidesma sp.* | Rhizophoraceae/ Malpighiales | BABRU3801-15 | MF435490 | n/a | 20-5493 |  |
| H290_APORS3 | *Aporosa sp.* | Rhizophoraceae/ Malpighiales | BABRU2348-15 | MF435494 | MF419009 | 19-0267 |  |
| H291_APORS1 | *Aporosa sp.* | Rhizophoraceae/ Malpighiales | BABRU4065-15 | MF435491 | n/a | 24-5761 |  |
| H292_APORS2 | *Aporosa sp.* | Rhizophoraceae/ Malpighiales | BABRU3694-15 | MF435493 | MF419012 | 20-5356 |  |
| H293_APORS4 | *Aporosa sp.* | Rhizophoraceae/ Malpighiales | BABRU4185-15 | MF435495 | MF419007 | 24-5890 |  |
| H294_APORAC | *Aporosa sp.* | Rhizophoraceae/ Malpighiales | BABRU3220-15 | MF435492 | MF419006 | 16-4523 |  |
| H295_APORBE | *Aporosa sp.* | Rhizophoraceae/ Malpighiales | BABRU2537-15 | MF435498 | MF419010 | 22-1043 |  |
| H296_APORBU | *Aporosa sp.* | Rhizophoraceae/ Malpighiales | BABRU3663-15 | MF435496 | n/a | 20-5323 |  |
| H297_APORSA | *Aporosa sp.* | Rhizophoraceae/ Malpighiales | BABRU2060-15 | MF435496 | MF419008 | 22-0350 |  |
| H298_APORSU1 | *Aporosa sp.* | Rhizophoraceae/ Malpighiales | BABRU1485-15 | MF435500 | MF419011 | 16-2460 |  |
| H299_APORSU2 | *Aporosa sp.* | Rhizophoraceae/ Malpighiales | BABRU1308-15 | MF435501 | n/a | 18-3133 |  |
| H300_BACCS1 | *Baccaurea sp.* | Rhizophoraceae/ Malpighiales | BABRU4003-15 | MF435502 | MF419013 | 24-5691 |  |
| H301_BACCS2 | *Baccaurea sp.* | Rhizophoraceae/ Malpighiales | BABRU2358-15 | MF435507 | MF419017 | 19-0277 |  |
| H302a_BACCS3 | *Baccaurea sp.* | Rhizophoraceae/ Malpighiales | BABRU2501-15 | MF435503 | MF419014 | 13-0884 |  |
| H302b_BACCRA | *Baccaurea sp.* | Rhizophoraceae/ Malpighiales | BABRU2439-15 | MF435505 | MF419016 | 25-0333 |  |
| H303_BACCCO | *Baccaurea sp.* | Rhizophoraceae/ Malpighiales | BABRU024-14 | MF435506 | MF419019 | 05-5375 |  |
| H304_BACCLA | *Baccaurea sp.* | Rhizophoraceae/ Malpighiales | BABRU3530-15 | MF435508 | MF419015 | 20-3764 |  |
| H305_BACCPY | *Baccaurea sp.* | Rhizophoraceae/ Malpighiales | BABRU4017-15 | MF435509 | MF419018 | 24-5706 |  |
| H306_BACCSA | *Baccaurea sp.* | Rhizophoraceae/ Malpighiales | BABRU1281-15 | MF435504 | n/a | 18-3104 |  |
| H307_CLEIHI | *Cleisthanthus sp.* | Rhizophoraceae/ Malpighiales | BABRU2493-15 | MF435510 | MF419021 | 13-0876 |  |
| H308_GLOCSP | *Glochidion sp.* | Rhizophoraceae/ Malpighiales | BABRU2273-15 | MF435513 | MF419023 | 24-0521 |  |
| H309_PHYLSP | *Cleisthanthus sp.* | Rhizophoraceae/ Malpighiales | BABRU1754-15 | MF435511 | MF419022 | 25-1863 |  |
| H310_DRYSP1 | *Drypetes sp.* | Putranjivaceae/ Malpighiales | BABRU1637-15 | MF435515 | MF418991 | 22-2130 |  |
| H311_DRYPER | *Drypetes sp.* | Putranjivaceae/ Malpighiales | BABRU901-14 | MF435514 | MF418987 | 04-4081 |  |
| H312_DRYPFA | *Drypetes sp.* | Putranjivaceae/ Malpighiales | BABRU050-14 | MF435516 | MF435872 | 05-5404 |  |
| H313_DRYPLO | *Drypetes sp.* | Putranjivaceae/ Malpighiales | BABRU1874-15 | n/a | MF418990 | 13-1878 |  |
| H314_DRYPMY | *Drypetes sp.* | Putranjivaceae/ Malpighiales | BABRU2381-15 | n/a | MF418989 | 25-0012 |  |
| H315_DRYPPO | *Drypetes sp.* | Putranjivaceae/ Malpighiales | BABRU868-14 | MF435517 | MF418988 | 04-4007 |  |
| H316_CASEGR | *Casearia sp.* | Salicaceae/ Malpighiales | BABRU4216-15 | MF435518 | MF419001 | 24-5921 |  |
| H317_FLACRU | *Flacourtia sp.* | Salicaceae/ Malpighiales | BABRU854-14 | MF435519 | MF418998 | 06-5360 |  |
| H318_HOMAIN | *Homalium sp.* | Salicaceae/ Malpighiales | BABRU2404-15 | MF435523 | MF419000 | 25-0035 |  |
| H319_SCOLSP | *Scolopia spinosa* | Salicaceae/ Malpighiales | BABRU134-14 | MF435524 | MF418999 | 10-5225 |  |
| H320_RINOS1 | *Rinorea sp.* | Violaceae/ Malpighiales | BABRU2949-15 | MF435525 | MF418993 | 24-3909 |  |
| H321_RINOS2 | *Rinorea sp.* | Violaceae/ Malpighiales | BABRU2166-15 | MF435526 | MF418994 | 15-0414 |  |
| H322_RINOS3 | *Rinorea sp.* | Violaceae/ Malpighiales | BABRU1738-15 | MF435529 | MF418995 | 25-1847 |  |
| H323_RINOHO | *Rinorea sp.* | Violaceae/ Malpighiales | BABRU815-14 | MF435527 | MF418997 | 01-5540 |  |
| H324_RINOLO | *Rinorea sp.* | Violaceae/ Malpighiales | BABRU3086-15 | MF435528 | MF418996 | 24-4051 |  |
| H325_ANISLA | *Anisoptera sp.* | Dipterocarpaceae/ Malvales | BABRU1400-15 | MF435530 | MF418805 | 13-3518 |  |
| H326_DIPTCASP | *Dipterocarpus sp.* | Dipterocarpaceae/ Malvales | BABRU1378-15 | MF435531 | MF418855 | 13-3495 |  |
| H327a_DIPTPASB | *Dipterocarpus sp.* | Dipterocarpaceae/ Malvales | BABRU1571-15 | MF435532 | MF418856 | 20-2907 |  |
| H327b_DIPTCA | *Dipterocarpus sp.* | Dipterocarpaceae/ Malvales | BABRU2178-15 | MF435533 | MF418857 | 15-0427 |  |
| H328_DRYOBE | *Dryobalanops sp.* | Dipterocarpaceae/ Malvales | BABRU229-14 | MF435535 | MF418853 | 14-4350 |  |
| H329_DRYOLA | *Dryobalanops sp.* | Dipterocarpaceae/ Malvales | BABRU810-14 | MF435536 | MF418854 | 01-5535 |  |
| H330_HOPEBR | *Hopea sp.* | Dipterocarpaceae/ Malvales | BABRU1357-15 | MF435537 | MF418847 | 13-3473 |  |
| H331_HOPDR | *Hopea sp.* | Dipterocarpaceae/ Malvales | BABRU2282-15 | MF435538 | MF418849 | 24-0530 |  |
| H332_HOPDR1 | *Hopea sp.* | Dipterocarpaceae/ Malvales | BABRU300-14 | MF435539 | MF418850 | 12-5263 |  |
| H333_HOPENE | *Hopea sp.* | Dipterocarpaceae/ Malvales | BABRU1161-14 | MF435540 | MF418848 | 04-4865 |  |
| H334_PARAMAC | *Parashorea* | Dipterocarpaceae/ Malvales | \|  \| BABRU4229-17 \| \| --- \| --- \| | MF435809 | MF435889 | 24-4723 |  |
| H335_PARAMA | *Parashorea* | Dipterocarpaceae/ Malvales | BABRU2401-15 | MF435541 | MF418815 | 25-0032 |  |
| H336_PARATO | *Parashorea* | Dipterocarpaceae/ Malvales | BABRU3603-15 | MF435542 | MF418814 | 20-3841 |  |
| H337a_SHORSP1 | *Shorea sp.* | Dipterocarpaceae/ Malvales | BABRU3446-15 | MF435545 | MF418845 | 20-3678 |  |
| H337b_SHORFAGUE_2 | *Shorea sp.* | Dipterocarpaceae/ Malvales | BABRU3720-15 | MF435549 | MF418841 | 20-5391 |  |
| H338_SHORSP2 | *Shorea sp.* | Dipterocarpaceae/ Malvales | BABRU304-14 | MF435551 | MF418843 | 12-5270 |  |
| H339a_SHORPAL | *Shorea sp.* | Dipterocarpaceae/ Malvales | BABRU3870-15 | MF435578 | n/a | 20-5582 |  |
| H339b_SHORPAU | *Shorea sp.* | Dipterocarpaceae/ Malvales | BABRU2573-15 | MF435579 | n/a | 22-1080 |  |
| H340_SHORSP2 | *Shorea sp.* | Dipterocarpaceae/ Malvales | BABRU967-14 | MF435548 | MF418840 | 04-4651 |  |
| H341_SHORSP4 | *Shorea sp.* | Dipterocarpaceae/ Malvales | BABRU1609-15 | MF435565 | MF418844 | 20-2946 |  |
| H342_SHORSP5 | *Shorea sp.* | Dipterocarpaceae/ Malvales | BABRU1096-14 | MF435570 | MF418838 | 01-3922 |  |
| H343_SHORSP9 | *Shorea sp.* | Dipterocarpaceae/ Malvales | BABRU2833-15 | MF435580 | MF418835 | 16-2932 |  |
| H344a_SHOAG | *Shorea sp.* | Dipterocarpaceae/ Malvales | BABRU4230-17 | MF435801 | MF435888 | 05-4419 |  |
| H344b_SHORCO | *Shorea sp.* | Dipterocarpaceae/ Malvales | BABRU4231-17 | MF435557 | MF418851 | 25-4813 |  |
| H345_SHORAM | *Shorea sp.* | Dipterocarpaceae/ Malvales | BABRU2097-15 | MF435553 | MF418837 | 15-0344 |  |
| H346_SHORBA | *Shorea sp.* | Dipterocarpaceae/ Malvales | BABRU053-14 | MF435544 | MF418839 | 05-5407 |  |
| H347_SHORBECC | *Shorea sp.* | Dipterocarpaceae/ Malvales | BABRU3151-15 | MF435543 | MF418825 | 16-4452 |  |
| H348_SHORBI | *Shorea sp.* | Dipterocarpaceae/ Malvales | BABRU3881-15 | MF435555 | MF418823 | 20-5597 |  |
| H349_SHOREX | *Shorea sp.* | Dipterocarpaceae/ Malvales | BABRU376-14 | MF435554 | MF418821 | 08-5668 |  |
| H350_SHORASA | *Shorea sp.* | Dipterocarpaceae/ Malvales | BABRU1414-15 | MF435552 | MF418816 | 13-2636 |  |
| H351_SHORFAGT | *Shorea sp.* | Dipterocarpaceae/ Malvales | BABRU2601-15 | n/a | MF435887 | 22-1109 |  |
| H352_SHORFAGU_1 | *Shorea sp.* | Dipterocarpaceae/ Malvales | BABRU3883-15 | MF435556 | MF418842 | 20-5601 |  |
| H353_SHORDO | *Shorea sp.* | Dipterocarpaceae/ Malvales | BABRU355-14 | MF435558 | MF418822 | 12-5324 |  |
| H354_SHORFA | *Shorea sp.* | Dipterocarpaceae/ Malvales | BABRU2962-15 | MF435559 | MF418834 | 24-3922 |  |
| H355_SHORHA1 | *Shorea sp.* | Dipterocarpaceae/ Malvales | BABRU2753-15 | MF435568 | MF418818 | 16-3007 |  |
| H356_SHORHA2 | *Shorea sp.* | Dipterocarpaceae/ Malvales | BABRU2783-15 | MF435569 | MF418819 | 16-3040 |  |
| H357_SHORAT | *Shorea sp.* | Dipterocarpaceae/ Malvales | BABRU966-14 | MF435563 | MF418824 | 04-4650 |  |
| H358a_SHORLE | *Shorea sp.* | Dipterocarpaceae/ Malvales | BABRU4048-15 | MF435571 | MF418829 | 24-5744 |  |
| H358b_SHORSP6 | *Shorea sp.* | Dipterocarpaceae/ Malvales | BABRU4232-17 | MF435573 | MF418831 | 19-1085 |  |
| H358c_SHORSC | *Shorea sp.* | Dipterocarpaceae/ Malvales | BABRU4043-15 | MF435572 | MF418828 | 24-5738 |  |
| H358d_SHORFE | *Shorea sp.* | Dipterocarpaceae/ Malvales | BABRU1478-15 | MF435574 | MF418832 | 16-2452 |  |
| H358e_SHORMASB | *Shorea sp.* | Dipterocarpaceae/ Malvales | BABRU2933-15 | MF435575 | MF418827 | 24-3893 |  |
| H359_SHORMASB2 | *Shorea sp.* | Dipterocarpaceae/ Malvales | BABRU004-14 | MF435576 | MF418833 | 05-5346 |  |
| H360_SHORMASM | *Shorea sp.* | Dipterocarpaceae/ Malvales | BABRU3358-15 | MF435577 | MF418830 | 16-4666 |  |
| H361_SHORMA | *Shorea sp.* | Dipterocarpaceae/ Malvales | BABRU1545-15 | MF435560 | MF418817 | 20-2879 |  |
| H362_SHORGI | *Shorea sp.* | Dipterocarpaceae/ Malvales | BABRU2774-15 | MF435560 | MF418846 | 16-3031 |  |
| H363_SHOROB | *Shorea sp.* | Dipterocarpaceae/ Malvales | BABRU4035-15 | MF435562 | MF418820 | 24-5730 |  |
| H364_SHOROC | *Shorea sp.* | Dipterocarpaceae/ Malvales | BABRU1627-15 | MF435564 | MF418852 | 22-2120 |  |
| H365_SHORPARSVE | *Shorea sp.* | Dipterocarpaceae/ Malvales | BABRU1916-15 | MF435566 | MF418826 | 25-2700 |  |
| H366_SHORPAT | *Shorea sp.* | Dipterocarpaceae/ Malvales | BABRU984-14 | MF435547 | n/a | 04-4670 |  |
| H367_SHORPI | *Shorea sp.* | Dipterocarpaceae/ Malvales | BABRU327-14 | MF435567 | MF418836 | 12-5295 |  |
| H568_SHORLA | *Shorea sp.* | Dipterocarpaceae/ Malvales | BABRU4233-17 | MF435807 | MF435886 | 05-3814 |  |
| H369_VATISP2 | *Vatica sp.* | Dipterocarpaceae/ Malvales | BABRU4196-15 | MF435587 | MF418812 | 24-5901 |  |
| H370_VATIDU | *Vatica sp.* | Dipterocarpaceae/ Malvales | BABRU1355-15 | MF435583 | MF418813 | 13-3471 |  |
| H371_VATIEN | *Vatica sp.* | Dipterocarpaceae/ Malvales | BABRU1275-14 | MF435582 | MF418806 | 04-4496 |  |
| H372_VATIMI | *Vatica sp.* | Dipterocarpaceae/ Malvales | BABRU1629-15 | MF435584 | MF418807 | 22-2122 |  |
| H373_VATINI | *Vatica sp.* | Dipterocarpaceae/ Malvales | BABRU1695-15 | MF435589 | MF418809 | 22-1846 |  |
| H374_VATIOBOB | *Vatica sp.* | Dipterocarpaceae/ Malvales | BABRU2114-15 | MF435585 | MF418810 | 15-0361 |  |
| H375_VATIOD | *Vatica sp.* | Dipterocarpaceae/ Malvales | BABRU4179-15 | MF435586 | MF418811 | 24-5883 |  |
| H376_VATISA | *Vatica sp.* | Dipterocarpaceae/ Malvales | BABRU3619-15 | MF435588 | MF418808 | 20-5275 |  |
| H377_DURIS | *Durio sp.* | Malvaceae/ Malvales | BABRU2626-15 | MF435594 | MF418785 | 22-1136 |  |
| H378_DURIGRA | *Durio sp.* | Malvaceae/ Malvales | BABRU1783-15 | MF435590 | n/a | 25-1893 |  |
| H379_DURIGR | *Durio sp.* | Malvaceae/ Malvales | BABRU1581-15 | MF435591 | MF418784 | 20-2917 |  |
| H380_DURIGRI | *Durio sp.* | Malvaceae/ Malvales | BABRU1590-15 | MF435593 | KU519689 | 20-2926 |  |
| H381_HERISI | *Heritiera sp.* | Malvaceae/ Malvales | BABRU2793-15 | MF435595 | MF418791 | 16-2892 |  |
| H382_LEPTCA | *Leptonychia sp.* | Malvaceae/ Malvales | BABRU3323-15 | MF435596 | MF418796 | 16-4631 |  |
| H383_MICRBL | *Microcos sp.* | Malvaceae/ Malvales | BABRU3416-15 | MF435597 | MF418797 | 20-3647 |  |
| H84_MICRFI | *Microcos sp.* | Malvaceae/ Malvales | BABRU870-14 | MF435601 | MF418800 | 04-4009 |  |
| H385a_MICRHI | *Microcos sp.* | Malvaceae/ Malvales | BABRU153-14 | MF435598 | MF418799 | 13-6050 |  |
| H385b_MICRLA | *Microcos sp.* | Malvaceae/ Malvales | BABRU1907-15 | MF435600 | MF418802 | 25-2685 |  |
| H385c_MICRCI | *Microcos sp.* | Malvaceae/ Malvales | BABRU1299-15 | MF435599 | MF418803 | 18-3123 |  |
| H386a_MICROS | *Microcos sp.* | Malvaceae/ Malvales | BABRU3680-15 | MF435602 | MF418798 | 20-5342 |  |
| H386b_MICRST | *Microcos sp.* | Malvaceae/ Malvales | BABRU260-14 | MF435603 | MF418801 | 12-4160 |  |
| H387_PENTAD | *Pentace sp.* | Malvaceae/ Malvales | BABRU1058-14 | MF435626 | MF418794 | 01-3862 |  |
| H388_PTERSU | *Pterospermum sp.* | Malvaceae/ Malvales | BABRU2480-15 | MF435604 | MF418795 | 23-0192 |  |
| H389_SCAPAL | *Scaphium sp.* | Malvaceae/ Malvales | BABRU1479-15 | MF435605 | MF418793 | 16-2453 |  |
| H390_SCAPMA | *Scaphium sp.* | Malvaceae/ Malvales | BABRU2362-15 | MF435607 | MF418792 | 19-0281 |  |
| H391_STERS | *Sterculia* sp. | Malvaceae/ Malvales | BABRU381-14 | MF435609 | MF435885 | 09-4140 |  |
| H392_STERCO | *Sterculia* sp. | Malvaceae/ Malvales | BABRU341-14 | MF435611 | MF418790 | 12-5309 |  |
| H393a_STERRY | *Sterculia* sp. | Malvaceae/ Malvales | BABRU1443-15 | MF435608 | MF41878 | 13-2666 |  |
| H393b_STERS1 | *Sterculia* sp. | Malvaceae/ Malvales | BABRU130-14 | MF435610 | MF418789 | 10-5220 |  |
| H394_STERRU | *Sterculia* sp. | Malvaceae/ Malvales | BABRU1411-15 | MF435613 | MF418787 | 13-2633 |  |
| H395_STERST | *Sterculia* sp. | Malvaceae/ Malvales | BABRU2118-15 | MF435612 | MF418788 | 15-0365 |  |
| H396_AQUILBE | *Aquilaria sp.* | Thymelaeaceae/ Malvales | BABRU2425-15 | MF435614 | MF418804 | 25-0317 |  |
| H397_AQILMI | *Aquilaria sp.* | Thymelaeaceae/ Malvales | BABRU3463-15 | MF435615 | n/a | 20-3695 |  |
| H398_GONYSP | *Gonystylus sp.* | Thymelaeaceae/ Malvales | BABRU430-14 | MF435616 | n/a | 09-4192 |  |
| H399_CRYPMA | *Crypteronia sp.* | Crypteroniaceae/ Myrtales | BABRU1619-15 | MF435617 | n/a | 22-2112 |  |
| H400_LIJNLA | Melastomataceae | Melastomataceae/ Myrtales | BABRU3303-15 | MF435619 | MF419175 | 16-4611 |  |
| H401_MEMEAR | *Memecylon sp.* | Melastomataceae/ Myrtales | BABRU413-14 | MF435620 | MF419176 | 09-4172 |  |
| H402_MEMECA | *Memecylon sp.* | Melastomataceae/ Myrtales | BABRU1104-14 | MF435804 | n/a | 03-5081 |  |
| H403_MEMEFL | *Memecylon sp.* | Melastomataceae/ Myrtales | BABRU1827-15 | MF435621 | n/a | 23-3285 |  |
| H404a_MEMELO | *Memecylon sp.* | Melastomataceae/ Myrtales | BABRU173-14 | MF435623 | n/a | 13-6070 |  |
| H404b_MEMEPA | *Memecylon sp.* | Melastomataceae/ Myrtales | BABRU3865-15 | MF435624 | n/a | 20-5576 |  |
| H405_MEMEME | *Memecylon sp.* | Melastomataceae/ Myrtales | BABRU3648-15 | MF435622 | n/a | 20-5306 |  |
| H406_OXYSBE | Melastomataceae | Melastomataceae/ Myrtales | BABRU1511-15 | MF435625 | MF419180 | 16-2489 |  |
| H407a_PTERS1 | *Pternandra sp.* | Melastomataceae/ Myrtales | BABRU971-14 | MF435627 | n/a | 04-4656 |  |
| H407b_PTERCR | *Pternandra sp.* | Melastomataceae/ Myrtales | BABRU2941-15 | MF435628 | n/a | 24-3901 |  |
| H408_PTERAZ | *Pternandra sp.* | Melastomataceae/ Myrtales | BABRU2926-15 | MF435631 | MF419178 | 24-3886 |  |
| H409_PTEREC | *Pternandra echinata* | Melastomataceae/ Myrtales | BABRU456-14 | MF435630 | MF419179 | 08-6444 |  |
| H410_PTERMU | *Pternandra sp.* | Melastomataceae/ Myrtales | BABRU2457-15 | MF435629 | MF419177 | 25-0351 |  |
| H411_SYZYSP | *Syzygium sp.* | Myrtaceae/ Myrtales | BABRU480-14 | MF435633 | MF418783 | 11-5705 |  |
| H412_SYZYS1 | *Syzygium sp.* | Myrtaceae/ Myrtales | BABRU2533-15 | MF435653 | MF418782 | 13-0921 |  |
| H413_SYZYS3 | *Syzygium sp.* | Myrtaceae/ Myrtales | BABRU2454-15 | MF435646 | MF418767 | 25-0348 |  |
| H414_SYZYS4 | *Syzygium sp.* | Myrtaceae/ Myrtales | BABRU1812-15 | MF435654 | MF418775 | 23-3269 |  |
| H415_SYZYS8 | *Syzygium sp.* | Myrtaceae/ Myrtales | BABRU3595-15 | MF435656 | MF418774 | 20-3832 |  |
| H416_SYZYS14 | *Syzygium sp.* | Myrtaceae/ Myrtales | BABRU2966-15 | MF435634 | MF418777 | 24-3926 |  |
| H417_SYZYS17 | *Syzygium sp.* | Myrtaceae/ Myrtales | BABRU2012-15 | MF435635 | MF418773 | 19-1933 |  |
| H418_SYZYS19 | *Syzygium sp.* | Myrtaceae/ Myrtales | BABRU017-14 | MF435636 | MF418766 | 05-5362 |  |
| H419_SYZYS22 | *Syzygium sp.* | Myrtaceae/ Myrtales | BABRU3954-15 | MF435637 | MF418771 | 24-5636 |  |
| H420_SYZYS23 | *Syzygium sp.* | Myrtaceae/ Myrtales | BABRU414-14 | MF435638 | MF418772 | 09-4173 |  |
| H421_SYZYS25 | *Syzygium sp.* | Myrtaceae/ Myrtales | BABRU4213-15 | MF435639 | MF418768 | 24-5918 |  |
| H422_SYZYS28 | *Syzygium sp.* | Myrtaceae/ Myrtales | BABRU3554-15 | MF435640 | n/a | 20-3789 |  |
| H423_SYZYS29 | *Syzygium sp.* | Myrtaceae/ Myrtales | BABRU2828-15 | MF435645 | MF418769 | 16-2927 |  |
| H424_SYZYS30 | *Syzygium sp.* | Myrtaceae/ Myrtales | BABRU1211-14 | MF435647 | n/a | 04-4997 |  |
| H425_SYZYS32 | *Syzygium sp.* | Myrtaceae/ Myrtales | BABRU2217-15 | MF435649 | MF418770 | 16-0886 |  |
| H426_SYZYS33 | *Syzygium sp.* | Myrtaceae/ Myrtales | BABRU3608-15 | MF435650 | MF418778 | 20-3846 |  |
| H427_SYZYS34 | *Syzygium sp.* | Myrtaceae/ Myrtales | BABRU3487-15 | MF435651 | MF418780 | 20-3719 |  |
| H428_SYZYS38 | *Syzygium sp.* | Myrtaceae/ Myrtales | BABRU2712-15 | MF435652 | MF418781 | 16-3085 |  |
| H429a_SYZYS39 | *Syzygium sp.* | Myrtaceae/ Myrtales | BABRU131-14 | MF435641 | MF418776 | 10-5222 |  |
| H429b_SYZYS40 | *Syzygium sp.* | Myrtaceae/ Myrtales | BABRU3784-15 | MF435642 | MF418764 | 20-5473 |  |
| H429c_SYZYS41 | *Syzygium sp.* | Myrtaceae/ Myrtales | BABRU3650-15 | MF435643 | MF418763 | 20-5308 |  |
| H429d_SYZYS15 | *Syzygium sp.* | Myrtaceae/ Myrtales | BABRU2308-15 | MF435644 | MF418765 | 19-0227 |  |
| H430SYZYS42 | *Syzygium sp.* | Myrtaceae/ Myrtales | BABRU2716-15 | MF435655 | MF418779 | 16-3089 |  |
| H431_CONAS1 | *Connaraceae sp.* | Connaraceae/ Oxalidales | BABRU3788-15 | MF435657 | MF418942 | 20-5477 |  |
| H232_CONAS2 | *Connaraceae sp.* | Connaraceae/ Oxalidales | BABRU3233-15 | MF435658 | MF418943 | 16-4536 |  |
| H433_ELAECL | *Elaeocarpus sp.* | Elaeocarpaceae/ Oxalidales | BABRU1868-15 | MF435661 | n/a | 13-1872 |  |
| H434_ELAENI | *Elaeocarpus sp.* | Elaeocarpaceae/ Oxalidales | BABRU1388-15 | MF435662 | MF418944 | 13-3506 |  |
| H435_SLOAJA | *Sloanea sp.* | Elaeocarpaceae/ Oxalidales | BABRU094-14 | MF435663 | n/a | 09-3686 |  |
| H436_HELIPE | *Helicia sp.* | Proteaceae/ Proteales | BABRU3754-15 | MF435664 | n/a | 20-5434 |  |
| H437_MELISP | *Meliosma sp.* | Sabiaceae/ Proteales | BABRU946-14 | MF435665 | n/a | 04-4629 |  |
| H438_MELISU | *Meliosma sp.* | Sabiaceae/ Proteales | BABRU3379-15 | MF435666 | MF419144 | 20-3610 |  |
| H439_GIRONE | *Gironniera sp.* | Cannabaceae/ Rosales | BABRU3210-15 | MF435667 | MF419043 | 16-4513 |  |
| H440_GIROSU | *Gironniera sp.* | Cannabaceae/ Rosales | BABRU3725-15 | MF435668 | MF419044 | 20-5397 |  |
| H441_ARTOS1 | *Artocarpus sp.* | Moraceae/ Rosales | BABRU1980-15 | MF435677 | MF419030 | 19-1901 |  |
| H442_ARTOS2 | *Artocarpus sp.* | Moraceae/ Rosales | BABRU3987-15 | MF435678 | MF419031 | 24-5672 |  |
| H443_ARTOAN | *Artocarpus sp.* | Moraceae/ Rosales | BABRU3737-15 | MF435669 | MF419025 | 20-5415 |  |
| H444_ARTOIN | *Artocarpus sp.* | Moraceae/ Rosales | BABRU1563-15 | MF435670 | MF419032 | 20-2898 |  |
| H445_ARTONI | *Artocarpus sp.* | Moraceae/ Rosales | BABRU2036-15 | MF435671 | MF419024 | 19-1962 |  |
| H446_ARTOKE | *Artocarpus sp.* | Moraceae/ Rosales | BABRU1139-14 | MF435673 | MF419029 | 03-5117 |  |
| H447_ARTOME | *Artocarpus sp.* | Moraceae/ Rosales | BABRU3868-15 | MF435674 | MF419026 | 20-5579 |  |
| H448_ARTOOD1 | *Artocarpus sp.* | Moraceae/ Rosales | BABRU2196-15 | MF435675 | MF419028 | 16-0864 |  |
| H449_ARTOOD2 | *Artocarpus sp.* | Moraceae/ Rosales | BABRU3869-15 | MF435676 | MF419027 | 20-5580 |  |
| H450_ARTOTA | *Artocarpus sp.* | Moraceae/ Rosales | BABRU3925-15 | MF435679 | n/a | 24-5599 |  |
| H451_FICUSSP1 | *Ficus sp.* | Moraceae/ Rosales | BABRU1228-14 | MF435686 | MF419037 | 11-4084 |  |
| H452_FICUS2 | *Ficus sp.* | Moraceae/ Rosales | BABRU4121-15 | MF435687 | MF419040 | 24-5817 |  |
| H453_FICUS3 | *Ficus sp.* | Moraceae/ Rosales | BABRU2284-15 | MF435688 | MF419038 | 24-0532 |  |
| H454_FICUS4 | *Ficus sp.* | Moraceae/ Rosales | BABRU853-14 | MF435689 | MF419035 | 06-5359 |  |
| H455_FICUS5 | *Ficus sp.* | Moraceae/ Rosales | BABRU1663-15 | MF435690 | MF419041 | 22-2159 |  |
| H456_FICUS6 | *Ficus sp.* | Moraceae/ Rosales | BABRU3981-15 | MF435691 | MF419042 | 24-5665 |  |
| H457_FICUAU | *Ficus sp.* | Moraceae/ Rosales | BABRU1179-14 | MF435680 | MF419033 | 04-4931 |  |
| H458a_FICUBE | *Ficus sp.* | Moraceae/ Rosales | BABRU373-14 | MF435681 | MF419036 | 08-5664 |  |
| H458b_FICUME | *Ficus sp.* | Moraceae/ Rosales | BABRU448-14 | MF435682 | MF419036 | 08-6436 |  |
| H459_FICUSE | *Ficus sp.* | Moraceae/ Rosales | BABRU1239-14 | MF435683 | MF419034 | 11-4096 |  |
| H460_RHAMSP | *Ziziphus angustifolius* | Rhamnaceae/ Rosales | BABRU788-14 | MF435694 | MF419046 | 01-5417 |  |
| H461_ZIZIS1 | *Ziziphus sp.* | Rhamnaceae/ Rosales | BABRU1300-15 | MF435695 | MF419045 | 18-3124 |  |
| H462_PRUNGR | *Prunus sp.* | Rosaceae/ Rosales | BABRU3601-15 | MF435806 | n/a | 20-3839 |  |
| H463_ANACFR | *Anacolosa sp.* | Olacaceae/ Santalales | BABRU883-14 | MF435696 | MF418750 | 04-4029 |  |
| H464_OCHAAM | *Ochanostachys amentacea* | Olacaceae/ Santalales | BABRU1108-14 | MF435699 | MF418663 | 03-5085 |  |
| H465_STROCE | *Strombosia sp.* | Olacaceae/ Santalales | BABRU1656-15 | MF435698 | KU519665 | 22-2151 |  |
| H466_OIPLX | *Opiliaceae sp.* | Opiliaceae/ Santalales | BABRU3310-15 | MF435700 | MF418751 | 16-4618 |  |
| H467_ANACSP | *Anacardiaceae sp.* | Anarcadiaceae/ Santalales | BABRU3921-15 | MF435701 | MF418858 | 24-5594 |  |
| H468_BUCHSE | Anarcadiaceae | Anarcadiaceae/ Santalales | BABRU2451-15 | MF435702 | MF418860 | 25-0345 |  |
| H469_CAMPAU | *Campnosperma sp.* | Anarcadiaceae/ Santalales | BABRU1230-14 | MF435703 | MF418859 | 11-4086 |  |
| H470_DRYMLU | Anarcadiaceae | Anarcadiaceae/ Santalales | BABRU2563-15 | MF435704 | MF418880 | 22-1070 |  |
| H471_GLUTLA | *Gluta sp.* | Anarcadiaceae/ Santalales | BABRU212-14 | MF435705 | MF418890 | 14-4333 |  |
| H472_GLUTMA | Anarcadiaceae | Anarcadiaceae/ Santalales | BABRU4025-15 | MF435707 | n/a | 24-5718 |  |
| H473_GLUTRU | *Gluta sp.* | Anarcadiaceae/ Santalales | BABRU2359-15 | MF435706 | MF418891 | 19-0278 |  |
| H474_GLUTWA | *Gluta sp.* | Anarcadiaceae/ Santalales | BABRU322-14 | MF435708 | MF418892 | 12-5289 |  |
| H475a_MELASP | Anarcadiaceae | Anarcadiaceae/ Santalales | BABRU2629-15 | MF435713 | MF418883 | 16-2944 |  |
| H475b_MELAS1 | Anarcadiaceae | Anarcadiaceae/ Santalales | BABRU795-14 | MF435714 | MF418884 | 01-5516 |  |
| H475c_MELAS2 | Anarcadiaceae | Anarcadiaceae/ Santalales | BABRU3831-15 | MF435715 | MF418885 | 20-5539 |  |
| H475d_MELABE | Anarcadiaceae | Anarcadiaceae/ Santalales | BABRU1811-15 | MF435710 | MF418881 | 23-3268 |  |
| H475e_MELABU | Anarcadiaceae | Anarcadiaceae/ Santalales | BABRU3984-15 | MF435711 | MF418882 | 24-5669 |  |
| H476_MELATO | Anarcadiaceae | Anarcadiaceae/ Santalales | BABRU119-14 | MF435712 | MF418889 | 10-5209 |  |
| H477_PARISP | *Parishia sp.* | Anarcadiaceae/ Santalales | BABRU2736-15 | MF435716 | MF418888 | 16-3109 |  |
| H478_SEMEEU | *Semecarpus sp.* | Anarcadiaceae/ Santalales | BABRU1943-15 | MF435718 | MF418887 | 16-1764 |  |
| H479_SEMERU | *Semecarpus sp.* | Anarcadiaceae/ Santalales | BABRU3993-15 | MF435717 | MF418886 | 24-5680 |  |
| H480_SWINFO | Anarcadiaceae | Anarcadiaceae/ Santalales | BABRU1311-15 | MF435719 | MF418893 | 18-3136 |  |
| H481_SWINGL | Anarcadiaceae | Anarcadiaceae/ Santalales | BABRU3894-15 | MF435720 | n/a | 20-5613 |  |
| H482_BURSXXI | *Burseracea sp.* | Burseraceae/ Sapindales | BABRU2661-15 | MF435721 | MF418861 | 16-2977 |  |
| H483_CANASP | Burseraceae | Burseraceae/ Sapindales | BABRU3862-15 | MF435723 | MF418878 | 20-5571 |  |
| H484_CANASP2 | Burseraceae | Burseraceae/ Sapindales | BABRU4212-15 | MF435724 | MF418876 | 24-5917 |  |
| H485_CANALI | Burseraceae | Burseraceae/ Sapindales | BABRU1900-15 | MF435722 | MF418875 | 25-2677 |  |
| H486_DACRCO | *Dacryodes sp.* | Burseraceae/ Sapindales | BABRU2127-15 | MF435725 | MF418863 | 15-0374 |  |
| H487_DACRIN | *Dacryodes sp.* | Burseraceae/ Sapindales | BABRU3799-15 | MF435727 | MF418877 | 20-5491 |  |
| H488_DACRLA | *Dacryodes sp.* | Burseraceae/ Sapindales | BABRU2598-15 | MF435730 | MF418864 | 22-1106 |  |
| H489_DACRYRO1 | *Dacryodes sp.* | Burseraceae/ Sapindales | BABRU1005-14 | MF435732 | MF418867 | 04-5263 |  |
| H490_DACRYRO2 | *Dacryodes sp.* | Burseraceae/ Sapindales | BABRU2595-15 | MF435733 | MF418868 | 22-1103 |  |
| H491_DACRRURU | *Dacryodes sp.* | Burseraceae/ Sapindales | BABRU1823-15 | MF435731 | MF418865 | 23-3281 |  |
| H492_DACRRU | *Dacryodes sp.* | Burseraceae/ Sapindales | BABRU3691-15 | MF435726 | MF418874 | 20-5353 |  |
| H493_DACRSP | *Dacryodes sp.* | Burseraceae/ Sapindales | BABRU2519-15 | MF435734 | MF418866 | 13-0904 |  |
| H494_SANTSP | Burseraceae | Burseraceae/ Sapindales | BABRU421-14 | MF435737 | MF418873 | 09-4183 |  |
| H495_SANTAP | Burseraceae | Burseraceae/ Sapindales | BABRU179-14 | MF435735 | MF418872 | 13-6076 |  |
| H496_SANTAPVA | Burseraceae | Burseraceae/ Sapindales | BABRU4083-15 | MF435736 | MF418862 | 24-5779 |  |
| H497_SANTGR | Burseraceae | Burseraceae/ Sapindales | BABRU877-14 | MF435738 | MF418869 | 04-4023 |  |
| H498_SANTOB | Burseraceae | Burseraceae/ Sapindales | BABRU1898-15 | MF435739 | MF418871 | 25-2675 |  |
| H499_SANTTO | Burseraceae | Burseraceae/ Sapindales | BABRU1417-15 | n/a | MF418870 | 13-2639 |  |
| H500_TRIOMA | Burseraceae | Burseraceae/ Sapindales | BABRU2565-15 | MF435741 | MF418879 | 22-1072 |  |
| H501_AGLASXI | *Aglaia sp.* | Meliaceae/ Sapindales | BABRU3900-15 | MF435742 | MF418894 | 24-5572 |  |
| H502_AGLASP | *Aglaia sp.* | Meliaceae/ Sapindales | BABRU3442-15 | MF435743 | MF418911 | 20-3674 |  |
| H503_AGLAS2 | *Aglaia sp.* | Meliaceae/ Sapindales | BABRU848-14 | MF435751 | MF418915 | 06-5353 |  |
| H504_AGLAS1 | *Aglaia sp.* | Meliaceae/ Sapindales | BABRU070-14 | MF435756 | MF418920 | 09-3660 |  |
| H505_AGLAS13 | *Aglaia sp.* | Meliaceae/ Sapindales | BABRU3136-15 | MF435757 | MF418898 | 16-4437 |  |
| H506_AGLAS7 | *Aglaia sp.* | Meliaceae/ Sapindales | BABRU1995-15 | MF435749 | MF418909 | 19-1916 |  |
| H507_AGLAS9 | *Aglaia sp.* | Meliaceae/ Sapindales | BABRU817-14 | MF435750 | MF418913 | 01-5542 |  |
| H508_AGLAS10 | *Aglaia sp.* | Meliaceae/ Sapindales | BABRU2386-15 | MF435745 | MF418908 | 25-0017 |  |
| H509_AGLAS12 | *Aglaia sp.* | Meliaceae/ Sapindales | BABRU1850-15 | MF435746 | MF418899 | 13-1853 |  |
| H510_AGLAS11 | *Aglaia sp.* | Meliaceae/ Sapindales | BABRU2422-15 | MF435747 | MF418896 | 25-0314 |  |
| H511_AGLAS15 | *Aglaia sp.* | Meliaceae/ Sapindales | BABRU2347-15 | MF435748 | MF435871 | 19-0266 |  |
| H512_AGLAS24 | *Aglaia sp.* | Meliaceae/ Sapindales | BABRU3848-15 | MF435753 | MF418897 | 20-5557 |  |
| H513_AGLAS4 | *Aglaia sp.* | Meliaceae/ Sapindales | BABRU4098-15 | MF435754 | MF418895 | 24-5794 |  |
| H514_AGLAS3 | *Aglaia sp.* | Meliaceae/ Sapindales | BABRU2290-15 | MF435755 | MF418906 | 24-0538 |  |
| H515_AGLASRU | *Aglaia sp.* | Meliaceae/ Sapindales | BABRU3684-15 | MF435758 | MF418905 | 20-5346 |  |
| H516_AGLASI | *Aglaia sp.* | Meliaceae/ Sapindales | BABRU4064-15 | MF435744 | MF418912 | 24-5760 |  |
| H517_CHISSP2 | Meliaceae | Meliaceae/ Sapindales | BABRU4066-15 | MF435765 | n/a | 24-5762 |  |
| H518_CHISSP4 | Meliaceae | Meliaceae/ Sapindales | BABRU3983-15 | MF435760 | MF418921 | 24-5668 |  |
| H519_CHISS5 | Meliaceae | Meliaceae/ Sapindales | BABRU1178-14 | MF435759 | MF418917 | 04-4930 |  |
| H520_DYSOSP | Meliaceae | Meliaceae/ Sapindales | BABRU1441-15 | MF435761 | MF418922 | 13-2664 |  |
| H521_DYSOS1 | Meliaceae | Meliaceae/ Sapindales | BABRU3851-15 | MF435762 | MF418910 | 20-5560 |  |
| H522_DYSOS3 | Meliaceae | Meliaceae/ Sapindales | BABRU2324-15 | MF435767 | MF418902 | 19-0243 |  |
| H523_DYSOS4 | Meliaceae | Meliaceae/ Sapindales | BABRU4032-15 | MF435771 | MF418900 | 24-5727 |  |
| H524_DYSOS5 | Meliaceae | Meliaceae/ Sapindales | BABRU3952-15 | MF435772 | MF418901 | 24-5634 |  |
| H525_DYSOS6 | Meliaceae | Meliaceae/ Sapindales | BABRU4234-17 | MF435770 | MF418903 | 24-3851 |  |
| H526_DYSOS8 | Meliaceae | Meliaceae/ Sapindales | BABRU1521-15 | MF435769 | MF418904 | 20-2855 |  |
| H527_DYSOS10 | Meliaceae | Meliaceae/ Sapindales | BABRU283-14 | MF435764 | MF418919 | 12-4183 |  |
| H528_DYSOS13 | Meliaceae | Meliaceae/ Sapindales | BABRU1003-14 | MF435763 | n/a | 04-5261 |  |
| H529_DYSOS14 | Meliaceae | Meliaceae/ Sapindales | BABRU2219-15 | MF435766 | MF418916 | 16-0888 |  |
| H530_LANSDO | *Lansium sp.* | Meliaceae/ Sapindales | BABRU1112-14 | MF435773 | MF418907 | 03-5089 |  |
| H531_MELIXXI | *Meliaceae* | Meliaceae/ Sapindales | BABRU022-14 | MF435774 | MF418914 | 05-5372 |  |
| H532_REINHU | *Meliaceae* | Meliaceae/ Sapindales | BABRU2760-15 | MF435775 | MF418918 | 16-3014 |  |
| H533_WALSSXI | *Walsura sp.* | Meliaceae/ Sapindales | BABRU3479-15 | MF435776 | MF418923 | 20-3711 |  |
| H534_WALSSXII | *Walsura sp.* | Meliaceae/ Sapindales | BABRU1469-15 | MF435777 | MF418925 | 16-2443 |  |
| H535_WALSSXIII | *Walsura sp.* | Meliaceae/ Sapindales | BABRU3339-15 | MF435778 | MF418924 | 16-4647 |  |
| H536_GLYCSP | *Glycosmis sp.* | Rutaceae/ Sapindales | BABRU2438-15 | MF435781 | MF418941 | 25-0332 |  |
| H537_GLYCMA | *Glycosmis sp.* | Rutaceae/ Sapindales | BABRU1763-15 | MF435779 | MF418940 | 25-1872 |  |
| H538_LEPIS3 | Sapindaceae | Sapindaceae/ Sapindales | BABRU2108-15 | MF435782 | MF418927 | 15-0355 |  |
| H539_LEPIS4 | Sapindaceae | Sapindaceae/ Sapindales | BABRU2061-15 | MF435783 | MF418939 | 22-0351 |  |
| H540_LEPIFR | Sapindaceae | Sapindaceae/ Sapindales | BABRU1613-15 | MF435784 | MF418938 | 20-2950 |  |
| H541_LEPITE | Sapindaceae | Sapindaceae/ Sapindales | BABRU879-14 | MF435785 | MF418928 | 04-4025 |  |
| H542_NEPHCU | *Nephelium sp.* | Sapindaceae/ Sapindales | BABRU3276-15 | MF435786 | MF418932 | 16-4583 |  |
| H543_NEPHSP | *Nephelium sp.* | Sapindaceae/ Sapindales | BABRU1982-15 | MF435787 | MF418933 | 19-1903 |  |
| H544_NEPHS3 | *Nephelium sp.* | Sapindaceae/ Sapindales | BABRU3818-15 | MF435789 | MF418929 | 20-5515 |  |
| H545_NEPHS4 | *Nephelium sp.* | Sapindaceae/ Sapindales | BABRU1103-14 | MF435788 | MF418936 | 03-5080 |  |
| H546_NEPHME | *Nephelium sp.* | Sapindaceae/ Sapindales | BABRU3604-15 | MF435794 | MF418934 | 20-3842 |  |
| H547_NEPHSU | *Nephelium sp.* | Sapindaceae/ Sapindales | BABRU3050-15 | MF435790 | MF418937 | 24-4015 |  |
| H548_NEPHUN | *Nephelium sp.* | Sapindaceae/ Sapindales | BABRU1987-15 | MF435793 | MF418935 | 19-1908 |  |
| H549_POMEPI | Sapindaceae | Sapindaceae/ Sapindales | BABRU818-14 | MF435795 | MF418931 | 01-5543 |  |
| H550_SAPISP2 | Sapindaceae | Sapindaceae/ Sapindales | BABRU897-14 | MF435796 | MF418930 | 04-4071 |  |
| H551_SIMASP | *Eurycoma* | Simaroubaceae/ Sapindales | BABRU2832-15 | MF435797 | MF418926 | 16-2931 |  |
| H552_ERYCCR | *Erycibe sp.* | Convolvulaceae/ Solanales | BABRU2396-15 | n/a | MF418761 | 25-0027 |  |
| H553_ERYCGL | *Erycibe sp.* | Convolvulaceae/ Solanales | BABRU3053-15 | MF435798 | MF418760 | 24-4018 |  |
| H554_ERYCST | *Erycibe sp.* | Convolvulaceae/ ^S^olanales | BABRU144-14 | MF435799 | MF418762 | 10-5236 |  |
| H555_LEEAIN | *Leea sp.* | Vitaceae/ Vitales | BABRU2074-15 | MF435800 | MF418710 | 22-0365 |  |

Morphological identities are available on request from Dr. Kamariah Abu Salim (Environmental and Life Sciences, Faculty of Science, University of Brunei Darussalam, Brunei Darussalam).
